# Nsp14 of SARS-CoV-2 inhibits mRNA processing and nuclear export by targeting the nuclear cap-binding complex

**DOI:** 10.1093/nar/gkad483

**Published:** 2023-06-01

**Authors:** Jun Katahira, Tatsuya Ohmae, Mayo Yasugi, Ryosuke Sasaki, Yumi Itoh, Tomoko Kohda, Miki Hieda, Masami Yokota Hirai, Toru Okamoto, Yoichi Miyamoto

**Affiliations:** Laboratory of Cellular Molecular Biology, Graduate School of Veterinary Sciences, Osaka Metropolitan University, 1-58 Rinku-Orai-kita, Izumisano, Osaka 598-8531, Japan; Laboratory of Cellular Molecular Biology, Graduate School of Veterinary Sciences, Osaka Metropolitan University, 1-58 Rinku-Orai-kita, Izumisano, Osaka 598-8531, Japan; Laboratory of Veterinary Public Health, Graduate School of Veterinary Sciences, Osaka Metropolitan University, 1-58 Rinku-Orai-kita, Izumisano, Osaka 598-8531, Japan; RIKEN Center for Sustainable Resource Science, Mass Spectrometry and Microscopy Unit, 1-7-22 Suehiro. Tsurumi, Yokohama, Kanagawa 230-0045, Japan; Institute for Advanced Co-Creation Studies, Research Institute for Microbial Diseases, Osaka University, 3-1 Yamadaoka, Suita, Osaka 565-0871, Japan; Laboratory of Veterinary Epidemiology, Graduate School of Veterinary Sciences, Osaka Metropolitan University, 1-58 Rinku-Orai-kita, Izumisano, Osaka 598-8531, Japan; Department of Medical Technology, Ehime Prefectural University of Health Sciences, 543 Tobe-Cho Takaoda, Iyo, Ehime791-2102, Japan; RIKEN Center for Sustainable Resource Science, Mass Spectrometry and Microscopy Unit, 1-7-22 Suehiro. Tsurumi, Yokohama, Kanagawa 230-0045, Japan; Institute for Advanced Co-Creation Studies, Research Institute for Microbial Diseases, Osaka University, 3-1 Yamadaoka, Suita, Osaka 565-0871, Japan; Laboratory of Nuclear Transport Dynamics, National Institutes of Biomedical Innovation, Health, and Nutrition (NIBIOHN), 7-6-8 Saito Asagi, Ibaraki, Osaka 567-0085, Japan

## Abstract

To facilitate selfish replication, viruses halt host gene expression in various ways. The nuclear export of mRNA is one such process targeted by many viruses. SARS-CoV-2, the etiological agent of severe acute respiratory syndrome, also prevents mRNA nuclear export. In this study, Nsp14, a bifunctional viral replicase subunit, was identified as a novel inhibitor of mRNA nuclear export. Nsp14 induces poly(A)^+^ RNA nuclear accumulation and the dissolution/coalescence of nuclear speckles. Genome-wide gene expression analysis revealed the global dysregulation of splicing and 3′-end processing defects of replication-dependent histone mRNAs by Nsp14. These abnormalities were also observed in SARS-CoV-2-infected cells. A mutation introduced at the guanine-N7-methyltransferase active site of Nsp14 diminished these inhibitory activities. Targeted capillary electrophoresis-mass spectrometry analysis (CE-MS) unveiled the production of N7-methyl-GTP in Nsp14-expressing cells. Association of the nuclear cap-binding complex (NCBC) with the mRNA cap and subsequent recruitment of U1 snRNP and the stem-loop binding protein (SLBP) were impaired by Nsp14. These data suggest that the defects in mRNA processing and export arise from the compromise of NCBC function by N7-methyl-GTP, thus exemplifying a novel viral strategy to block host gene expression.

## Graphical Abstract

**Graphical Abstract:**
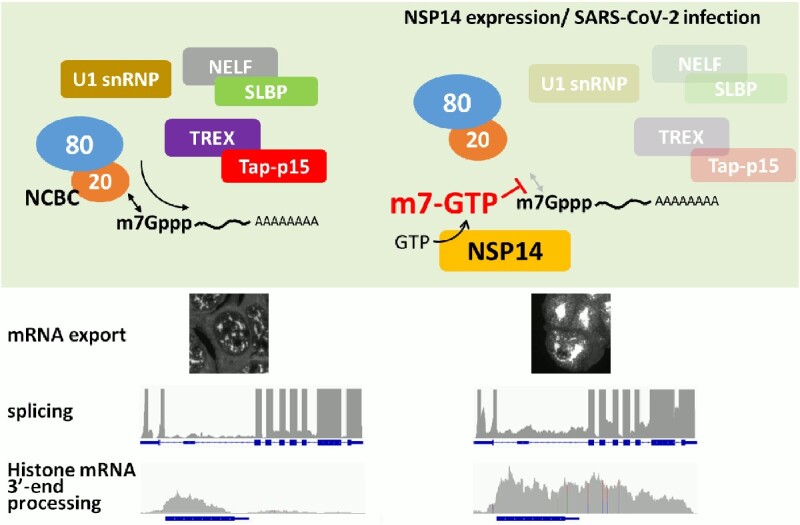

## INTRODUCTION

Since eukaryotic cells are compartmentalized to the nucleus and the cytoplasm, newly synthesized mRNAs must be transported to the cytoplasm for decoding genetic information by the translation machinery (1,2). Packaging of a mature mRNA into a messenger ribonucleoprotein (mRNP) particle, which is a prerequisite for transport through the nuclear pore complex (NPC), occurs cotranscriptionally in the nucleus (3–5). The carboxy-terminal domain (CTD) of the largest subunit of RNA polymerase II (RNAPII) initially plays essential roles in this process. The CTD code, which consists of various combinations of posttranslational modifications of the heptapeptide repeats (YSPTSPS), such as the phosphorylation of specific serine residues, leads to the pertinent recruitment of different pre-mRNA processing/maturation factors to active genes (6–8). During transcription initiation phase, S_5_ of the CTD is phosphorylated and attracts the capping enzyme (mRNA guanylyltransferase; RNGTT), which adds an inverted guanosine moiety to the 5′-end of a nascent transcript by the triphosphatase and guanylyltransferase activities (9,10). The cap methyltransferase (RNA guanine N-7 methyltransferase; RNMT), in turn, is then recruited to the transcription initiation site and adds a methyl-group to the N7-position of the cap guanosine base, although in this process the interaction with the phosphorylated CTD is likely to be indirect (11).

Capped nascent pre-mRNA is then bound by the nuclear cap binding complex (NCBC) for transcriptional elongation and further processing. NCBC is a multifunctional heterodimer consisting of CBP20 and CBP80, of which the former directly binds to the N7-methylated cap structure (12–14). NCBC, along with the CTD, influences subsequent steps in mRNA metabolism through interactions with numerous factors (15). The downstream processes in which NCBC and specific interaction partners have been implicated are highly divergent and include the following: promoter clearance and productive transcription elongation with positive transcription elongation factor b (P-TEFb) (16), splicing with U1, U2 and U4/U6·U5 snRNPs (17,18), cleavage and polyadenylation with the 3′-end processing factors (19), stem−loop binding protein (SLBP)-mediated replication-dependent (RD) histone mRNA maturation with negative elongation factor (NELF) (20), nuclear export with the TRanscription-EXport complex (TREX) (21), and translation with eIF-4G (22) and CBC-dependent translation initiation factor (CTIF) (23). In addition to these broad functions on mRNA metabolism, NCBC determines the fates of the other RNAPII-transcribed RNAs: the intracellular transport of U snRNAs as well as a small subset of capped snoRNAs is regulated by NCBC-PHAX (24,25), whereas the degradation of short-lived RNAs such as promotor upstream transcripts (PROMPTs) is accelerated by NCBC-ZC3H18 (26).

Although detailed molecular mechanisms remain under active investigation (15), recent studies have suggested that NCBP3, which was originally identified as an alternative for CBP20 (27), participates in the nascent mRNP through interaction with NCBC and recruits TREX, thus playing an important role in licensing mRNPs for nuclear export (28,29). Eventually, the conserved mRNA nuclear export receptor Tap-p15 (also called NXF1-NXT1) recognizes the fully maturated mRNP as a transport cargo via interaction with ALYREF, an mRNA binding adaptor component of TREX, allowing translocation of the mRNP through the NPC (5,30).

Viruses often exploit and/or inhibit the host nuclear RNA export processes to evade host defense systems and to promote their own gene expression for selfish replication. Pathogenic lentiviruses, such as human immunodeficiency virus (HIV), use the Rev protein to seize the CRM1-mediated U snRNA export pathway for the expression of their unspliced mRNAs (31,32). Simple retroviruses, such as simian retrovirus type 1, have evolved the constitutive transport element (CTE) in their RNA genomes to hijack the Tap-p15-mediated mRNA export pathway (33–35). Herpes simplex virus ICP27 facilitates the export of viral intronless mRNAs by using the ALYREF/Tap-p15-mediated mRNA export pathway (36). The vesicular stomatitis virus (VSV) matrix (M) protein shuts down host mRNA export by inhibiting the nucleoporins Nup98-Rae1 at the NPC (37,38). ORF10 of Kaposi's sarcoma-associated herpesvirus also targets Nup98-Rae1 and inhibits the nuclear export of select cellular mRNAs (39). Poliovirus 2A protease blocks the nuclear export of mRNA, rRNA and U snRNAs by cleaving Nup98 (40).

Inhibition of cellular mRNA export by SARS-CoV-2, the causative agent of the present pandemic of severe acute respiratory syndrome, has also been noted as a mechanism to contend for the host defense system (41) (see Supplementary Figure S1). Recent studies have indicated that both the ORF6 and NSP1 proteins of SARS-CoV-2 target the mRNA export process by inhibiting Nup98-Rae1 and Tap-p15, respectively (42–44), although the latter mechanism could also be explained differently (41,45,46). In this study, we assessed 24 different SARS-CoV-2 proteins for their mRNA export inhibitory activities and added Nsp14 to the short list of viral mRNA export inhibitors. The data presented in this report indicate that increased 7-methyl-GTP (m7GTP), which is produced by Nsp14 and can act as a cap mimic, perturbs the functions of NCBC in the processing and nuclear export of mRNAs, thus demonstrating a novel viral strategy to block host cell gene expression.

## MATERIALS AND METHODS

### Antibodies

Antibodies against Tap and the human THO/TREX components have been previously described (47,48). Rabbit polyclonal antibodies against GFP, mouse IgG, CBP20, SLBP, and SARS-CoV-2 spike (S) protein, mouse monoclonal antibodies against ALYREF, SC35, β-actin, FLAG-peptide tag, Rho-1D4 tag, Strep-tag II and a rat monoclonal antibody against U1C were commercially acquired. Antibodies against SARS-CoV-2 Nsp14 and puromycin were obtained from MRC PPU Reagents and Services, University of Dundee and DSHB, University of Iowa, respectively. Horseradish peroxidase (HRP)-conjugated secondary antibodies were purchased from Bio-Rad, whereas Alexa- and DyLight-conjugated secondary antibodies were purchased from Thermo Fisher Scientific. HRP-conjugated anti-rat IgG was purchased from Abcam. The monoclonal antibody 38A1 against CBP80 (49) was a gift from Drs Hito Ohno and Ichiro Taniguchi of Kyoto University. Details of the primary antibodies used in this study are listed in Supplementary Table S1.

### Plasmid construction

Mammalian expression vectors of the SARS-CoV-2 proteins (50), a DOX-inducible mammalian expression vector pInducer20 (51), and a human ACE2 expression vector pLENTI_hACE2_HygR (52) were obtained from Addgene (listed in Supplementary Table S2). The mammalian expression vector pCMV-FLAG3 was constructed by replacing the GFP ORF of the pEGFP-C1 vector (Clontech) with synthetic double-stranded oligo DNA encoding a 1xFLAG peptide tag (MDYKDDDDK). Expression vectors for GFP-Nsp14 fusion proteins were constructed as follows. Complementary DNAs (cDNAs) encoding GFP and Nsp14 together with a bovine growth hormone polyadenylation signal (BGH pA) cassette from pcDNA3.1 (Invitrogen) were amplified by polymerase chain reaction (PCR) and inserted into the pENTR4 entry vector (Invitrogen) by using the In-Fusion HD cloning kit (Clontech). The resulting GFP-Nsp14-BGH pA cassette was transferred to the pInducer20 vector by the Gateway system (Invitrogen). The D^90^VE → AVA and D^331^ → A mutations were introduced by the Quick Change kit (Agilent). For transient expression experiments, cDNAs encoding wild-type and mutant Nsp14 proteins were inserted into the BspEI-SalI site of the pEGFP-C1 vector. To remove the GFP-tag, the pEGFP -Nsp14 vector was doubly digested with NheI-BspEI, and a synthetic double-stranded oligonucleotide encoding the initiation methionine was inserted. The protein expressed from the resulting vector is Nsp14 appended with only four amino acids (MSGA) at the amino-terminus. For the construction of DcpS expression vectors, a cDNA fragment encoding mouse DcpS (GenBank accession NM_027030) was amplified by PCR from a mouse 7-day embryo cDNA library (Clontech) and inserted into the pCMV-FLAG3 and pENTR4-GFP-BGHpA vectors by the In-Fusion HD cloning kit. Transfer of the GFP-DcpS-BGH pA cassette was carried out as described above, and the resulting plasmid was named pInducer20-GFP-DcpS. All the sequences were verified by Sanger sequencing. Detailed plasmid maps are available upon request.

### Cell culture, transfection, and establishment of a stable cell line

Human 293F (Invitrogen) and HeLa (ATCC) cells were cultured in Dulbecco's modified Eagle's medium supplemented with 10% fetal calf serum (DMEM-10% FCS) under a 5% CO_2_ atmosphere. Vero E6 cells were cultured in minimal essential medium supplemented with 10% fetal calf serum (MEM-10% FCS). Transfection of plasmids was carried out by using Effectene transfection reagent (Qiagen) according to the manufacturer's protocol. To establish DOX-inducible cell lines, pInducer20 vectors harboring GFP fusions of wild-type and D^331^→A Nsp14 were linearized by SfiI and transfected into 293F cells. At 48 h after transfection, the cells were reseeded into 10 cm dishes containing DMEM-10% FCS supplemented with 1.2 mg/ml G418 (Nacalai Tesque). After 10 days, cell colonies were manually picked, and the individual clones were screened for DOX-inducible expression of the fusion proteins under a fluorescence microscope. Clones named 293F_Nsp14_wt2 (wild-type) and 293F_Nsp14_AIG44 (D^331^→A) were used for further analysis. A 293F cell line stably expressing hACE2 was established essentially as described above, except that 400 μg/ml hygromycin was used instead of G418 for selection. The clones were screened for their hACE2 expression levels by Western blotting and IFA using an anti-Rho-1D4 tag and SARS-CoV-2 susceptibility (Supplementary Figure S9). A clone named 293F_hACE2_21 was used for further analysis. To establish GFP-DcpS-inducible cell lines, SfiI-digested pInducer20-GFP-DcpS was transfected into 293F_hACE2_21 cells. Cells harboring the hACE2 and GFP-DcpS expression vectors were selected by 1.2 mg/ml G418 and 400 μg/ml hygromycin and a cell line designated 293F_hACE2_DcpS_29 was used for further analysis. DOX-inducible expression of the fusion protein in each cell clone was confirmed as described above (Supplementary Figure S9).

### Viral infection

The SARS-CoV-2 strain JPN/TY/WK/521 was provided by the National Institute of Infectious Diseases, Tokyo, Japan. Viruses were propagated in a monolayer of Vero E6/TMPRSS2 cells cultured in DMEM-2% FCS. Monolayers of Vero E6, 293F_hACE2_21 or 293F_hACE2_DcpS_29 cells in DMEM-2% FCS were infected with viruses at the indicated multiplicity of infection. After 24 hr of incubation, the cells were processed for further analysis.

### Oligo-dT *in situ* hybridization

Fluorescent *in situ* hybridization (FISH) using a Cy3-labeled oligo-dT_50_ probe was carried out as previously described (53). For double staining with antibodies, fixed cells were first subjected to immunofluorescence analysis using the indicated antibodies, postfixed with 4% paraformaldehyde, and then probed with a Cy3-labeled oligo-dT_50_ probe.

### RNA-seq and data analysis

Total RNA was isolated from uninduced or induced 293F_Nsp14_wt2 cells by TRIzol reagent (Ambion). Library construction using the TruSeq stranded mRNA kit and sequencing by NovaSeq 6000 (Illumina) were carried out by Macrogen Japan Corp. Poly(A)^+^ RNA-seq data SRR13952621 (SARS-CoV-2 infected) and SRR13952627 (uninfected) (54), which were downloaded by fasterq-dump from the National Center for Biotechnology Information sequence read archive (NCBI SRA), were used for gene expression analysis of SARS-CoV-2-infected A549 cells. For the analysis of inhibition of telescripting, SRR9864939 (control morpholino-treated cells) and SRR9864940 (U1-antisense morpholino-treated cells) (55) were used. Adapter trimming and quality filtering of the 101 base paired-end reads were carried out by fastp (56). The passed reads were mapped to the human genome 19 (hg19) assembly by using HISAT2 (57) or STAR (58). Differentially expressed genes were mined by featureCounts (59). Mapping to different gene features, i.e. coding sequences (CDSs), introns, intergenic regions, and untranslated regions (UTRs), were carried out by gatk4 collectRnaSeqMetrics (60) using an hg19 refFlat file downloaded from the University of California Santa Cruz (UCSC) site. Intron retention ratios (IR ratio) at different introns were calculated by IRFinder (61) using the bam files generated by STAR aligner. Of 249325 introns analyzed automatically by the software, those with the ‘LowCover’ warning under both uninduced and induced conditions were removed. Of the remaining 79724 introns, the IR ratios of 93% (74262 introns) were in the range of 0 to 0.3. Thus, only these introns were included in the heatmap and the box plot to avoid low confidence data (61). IR ratio analysis of SARS-CoV-2-infected cells was carried out in the same manner. Box plots were made by BoxPlotR (62). Conversion of the bam files to CPM-normalized bigWig files was performed using Deeptools bamCoverage (63). Metagene analysis of 69 human replication-dependent histone genes was carried out by Deeptools computeMatrix reference-point using a custom GTF file generated from data downloaded from the UCSC table browser. Heatmaps were drawn by Deeptools plotHeatmap.

### Quantitative PCR (qPCR) and quantitative RT−PCR (qRT−PCR)

qPCR analysis was carried out by using Luna Universal qPCR Master Mix. Reaction conditions were as per the manufacturer's protocol.

For qRT−PCR analysis, RNA isolation was carried out as described above. The total RNA samples were pretreated with a Turbo DNA-free kit (Thermo Fischer Scientific) to remove contaminated genomic DNA. Reverse transcription reactions were carried out using LunaScript RT SuperMix with oligo-dT and random hexamers. qPCR was carried out as described above. Relative amounts of the targets were determined by the standard curve method. The nucleotide sequences of the qPCR primers used are listed in Supplementary Table S3. Data are presented as averages of three biological replicates with standard deviation. Statistical significance was verified by two-tailed Welch's *t* test.

### RNA immunoprecipitation assay (RIP)

Cells with or without DOX induction (1 × 6 cm dish/IP) were harvested and resuspended in 600 μl of RIPA buffer (10 mM Tris–HCl (pH 8.0)/100 mM NaCl/1 mM EDTA/0.5 mM EGTA/1% Triton X-100/0.1% deoxycholate/0.05% SDS) and disrupted by brief sonication. The insoluble materials were removed by centrifugation. An aliquot (100 μl) of the supernatant was removed, and total RNA was isolated by TRIzol reagent (input fraction). The remaining portions were mixed with Dynabeads ProteinG that had been preincubated with each antibody. After 7 h of incubation at 4°C, the beads were extensively washed with RIPA buffer, and bound materials were extracted by TRIzol reagent (IP fraction). Contaminated genomic DNA was removed by a Turbo DNA-Free Kit. qRT−PCR analysis was carried out as described above. RIP efficiency was calculated by dividing the amount of each RNA in the IP fraction by that in the corresponding input fraction. Data are presented as averages of at least three technical replicates with standard deviation. The nucleotide sequences of the qPCR primers used are listed in Supplementary Table S3.

### Capillary electrophoresis coupled with mass spectrometry (CE-MS)

CE-MS and multitarget analysis were carried out as previously described (64). The m7GTP standard was purchased from Sigma.

By comparing the area of the CE peak of the known amount of the standard with those of the test samples, m7GTP produced in 4.3 × 10^6^ Nsp14-expressing cells was estimated to be 954 ± 117 pmol. Assuming that a 293F cell is a sphere with a diameter of ∼15 μm (65), the cellular concentration of m7GTP was calculated to be ∼119 μM.

### UV crosslinking and crosslinking-immunoprecipitation (CLIP)

UV crosslinking was performed essentially as described previously (47). 293F_Nsp14 cells (3 × 10 cm dishes/condition) with or without DOX induction were used. The UV dose was 300 mJ/cm^2^. The cells were lysed in RIPA buffer, and the soluble fractions were adjusted to final concentrations of 1% SDS and 0.5 M LiCl, then heat denatured and subjected to oligo-dT cellulose (Wako pure chemicals) chromatography. After extensive washing with RIPA containing 1% SDS and 0.5 M LiCl, bound materials were eluted with TE buffer (10 mM Tris–HCl (pH 8.0)/1 mM EDTA). The samples were concentrated by ethanol precipitation, treated with 100 μg/ml RNase A, and analyzed by SDS−PAGE followed by Western blotting using the antibodies specified in the figure.

CLIP was carried out as follows. UV-irradiated cells were harvested and resuspended in RIPA buffer. The cells were disrupted by brief sonication, and insoluble materials were removed by centrifugation. An aliquot (100 μl) of the supernatant was removed and treated with proteinase K (250 μg/ml), and total RNA was isolated by TRIzol reagent (input fraction). The remaining proteins were mixed with Dynabeads ProteinG, which had been pretreated with each antibody. After 8 h of incubation at 4°C, the beads were extensively washed with RIPA buffer. The bound materials were eluted with 50 mM Tris–HCl (pH 8.0)/5 mM EDTA/1% SDS at 65°C and diluted 2 times with H_2_O. After proteinase K treatment, RNA was extracted by TRIzol reagent (IP fraction). Contaminated genomic DNA was removed by a Turbo DNA-Free Kit. qRT−PCR analysis was carried out as described above. CLIP efficiency was calculated by dividing the amount of each RNA in the IP fraction by that in the corresponding input fraction. Data are presented as averages of at least three technical replicates with standard deviation. Reproducibility was confirmed by two to three independent experiments. The nucleotide sequences of the qPCR primers used are listed in Supplementary Table S3.

### Miscellaneous

A ribbon diagram showing the 3D structure of the SARS-CoV-2 Nsp14 protein (PDB accession number: 7R2V) (66) was drawn by Chimera 1.15 (67).

Global protein synthesis was monitored by analyzing incorporation of puromycin into the nascent peptide chains according to a method described in (68). 293F_Nsp14_wt2 cells without or with DOX induction (48 hr) were pulse labeled with puromycin at the concentrations specified in the figure for 10 min and chased for 60 min. Whole cell extracts were subjected to SDS-PAGE followed by Coomassie brilliant blue staining or Western blotting. The monoclonal anti-puromycin antibody was used at the concentration of 0.5 μg/ml.

## RESULTS

### Identification of Nsp14 as a novel viral mRNA export inhibitor

**Figure 1.**
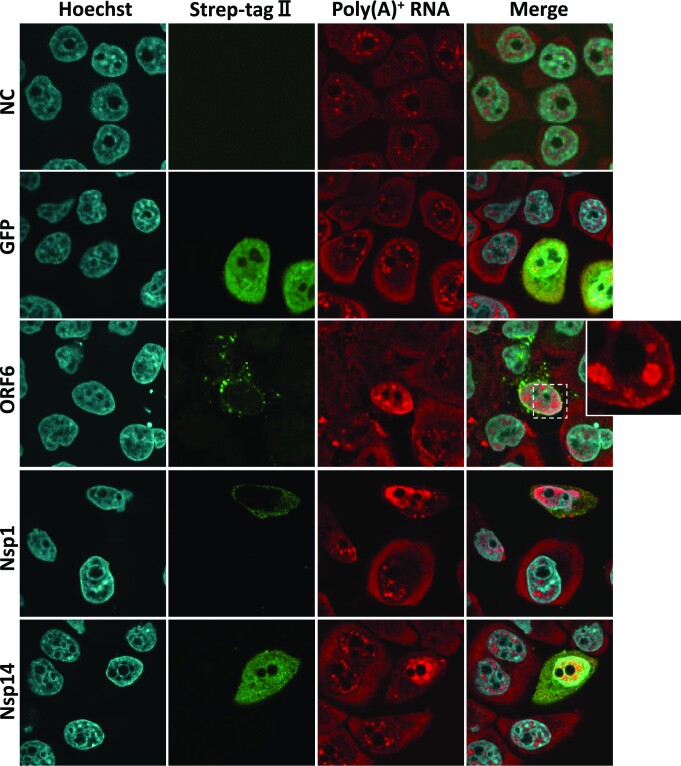
Identification of Nsp14 as a novel mRNA export inhibitor. HeLa cells cultured on glass coverslips were transfected with plasmids encoding GFP or the indicated viral proteins tagged with Strep-tag II. At 48 h after transfection, the cells were fixed and subjected to IFA with anti-Strept-tag II antibody followed by FISH using a Cy3-labeled oligo-dT_50_ probe. The cell nuclei were stained with Hoechst 33342. The cells were observed by confocal microscopy. Maximum-intensity projections of a single stack (10 consecutive slices, 0.35 μm *z*-distance) of images are shown. NC: nontransfected control.

Nuclear export of host cell mRNA was blocked upon SARS-CoV-2 infection (Supplementary Figure S1: SARS-CoV-2 infected Vero cells). To identify which viral proteins are responsible for export inhibition, we screened individual SARS-CoV-2 proteins for their nuclear export inhibitory activities by oligo-dT FISH analysis. Expression of the epitope-tagged viral proteins was readily detectable by immunofluorescence analysis (IFA) using an anti-tag antibody (Figure 1 and Supplementary Figure S2). Among the 24 different viral proteins tested, only three showed apparent export inhibitory activities (Figure 1, also see Supplementary Figure S2 for factors not affecting export). As previously reported, nuclear accumulation of poly(A)^+^ RNA was observed in cells expressing either ORF6 or Nsp1 proteins (42,43). In some instances, the FISH signals accumulated at the nuclear rim in ORF6-expressing cells (Figure 1, inset), which might reflect the disturbance of NPC function by the viral protein (42,50). In addition to these known factors, we found that Nsp14 strongly inhibits the nuclear export of bulk poly(A)^+^ RNAs (Figure 1).

**Figure 2.**
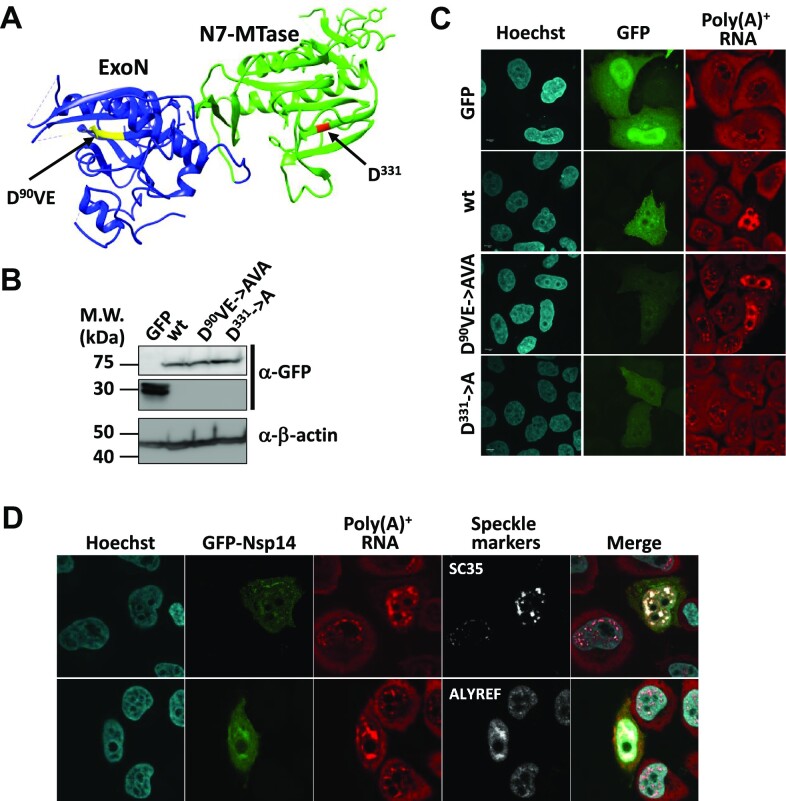
The N7-methyltransferase domain of SARS-CoV-2 Nsp14 is critical for nuclear mRNA export inhibition. (**A**) A ribbon diagram depicting the structure of the Nsp14 protein of SARS-CoV-2 (PDB: 7R2V). The protein consists of the N-terminal ExoN (blue) and the C-terminal N7-MTase (green) domains. The critical amino acid residues of each enzymatic activity are colored yellow and red. (**B**) HeLa cells were transfected with plasmids encoding GFP or GFP-fused with the wild-type and mutant Nsp14 proteins. The cells were harvested at 48 h after transfection, and whole-cell extracts prepared from each transfection were subjected to Western blotting using the indicated antibodies. (**C**) HeLa cells cultured on glass coverslips were transfected with plasmids encoding GFP or GFP-fused with the wild-type and mutant Nsp14 proteins. At 48 h after transfection, the cells were fixed and subjected to FISH using a Cy3-labeled oligo-dT_50_ probe. The cell nuclei were stained with Hoechst 33342. The cells were observed by confocal microscopy. Maximum-intensity projections of a single stack (10 consecutive slices, 0.35 μm z-distance) of images are shown. (**D**) HeLa cells cultured on glass coverslips were transfected with plasmids encoding GFP fused with the wild-type Nsp14 protein. At 48 h after transfection, the cells were fixed and subjected to IFA with anti-SC35 (upper) or anti-ALYREF (lower) antibodies followed by FISH using a Cy3-labeled oligo-dT_50_ probe. The cell nuclei were stained with Hoechst 33342. The cells were observed by confocal microscopy.

Nsp14 is a bifunctional viral replicase subunit consisting of the N-terminal exoribonuclease (ExoN) and the C-terminal guanine N7-methyltransferase (N7-MTase) domains (Figure 2A). Since the Nsp14 proteins from SARS-CoV and SARS-CoV-2 are almost identical (overall sequence identity is 95%), the previous data of SARS-CoV Nsp14 (69,70) could be extrapolated to SARS-CoV-2 Nsp14. Indeed, recent biochemical and structural studies revealed that the residues critical for the enzymatic activities are well conserved (71–73). Therefore, we selected two well-characterized mutants D^90^VE→AVA (ExoN mutant) and D^331^→A (N7-MTase mutant) to examine which enzymatic activity is required for poly(A)^+^ RNA export inhibition. The wild-type SARS-CoV-2 Nsp14 as well as the mutants were expressed as GFP fusion proteins. As shown by Western blotting, these mutant proteins were expressed as efficiently as the wild-type protein (Figure 2B). In transfected cells, they were localized in both the nucleus and the cytoplasm, which was indistinguishable from the localization of the wild-type protein. FISH analysis revealed that the D^90^VE→AVA mutant blocked poly(A)^+^ RNA export as efficiently as the wild-type protein, whereas the D^331^→A mutant showed almost complete loss of inhibitory activity (Figure 2C). These data indicate that the N7-MTase activity of Nsp14 is indispensable for the inhibition of the nuclear export of mRNAs. In Nsp14-expressing cells, poly(A)^+^ RNAs accumulated in nuclear speckle domains, and dissolution and/or coalescence of the speckles were observed (Figure 2D). Removal of most part of the GFP tag sequence did not affect the poly(A)^+^ RNA export inhibitory activity (Supplementary Figure S3), excluding a possibility that the addition of the tag at the N-terminus grossly altered the function of Nsp14.

### Nsp14 dysregulates pre-mRNA splicing and the 3′-end formation of replication dependent histone mRNAs

**Figure 3.**
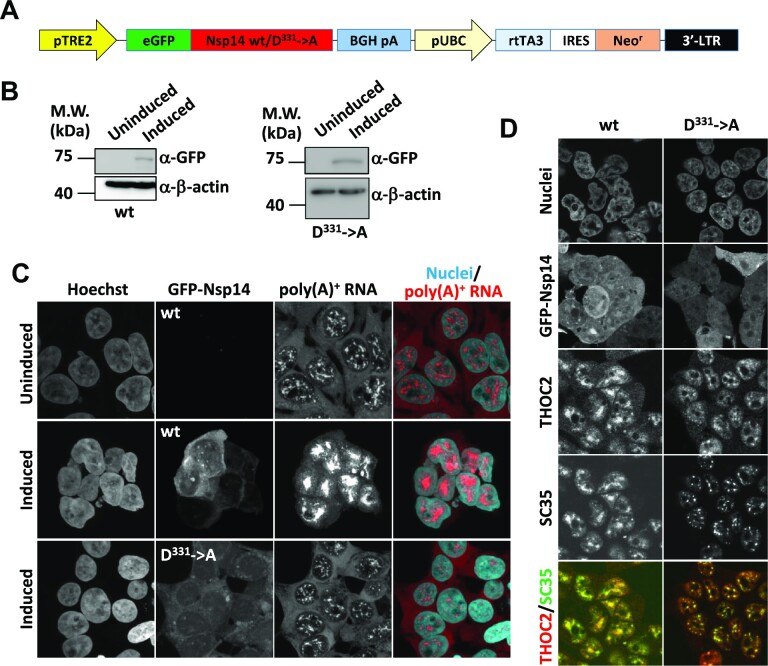
Establishment of DOX-inducible GFP-Nsp14 cell lines. (**A**) The structure of the expression plasmids. pTRE2: the modified tetracycline-response element and the CMV minimal promoter, eGFP: enhanced green fluorescent protein, BGH pA: polyadenylation signal from the bovine growth hormone gene, pUBC: the ubiquitin C promotor, rtTA3: reverse tetracycline transactivator 3, IRES: internal ribosome entry site, Neo^r^: neomycin resistance gene, 3′-LTR: 3′-long terminal repeat of HIV. (**B**) 293F_Nsp14_wt2 (left panels) and 293F_Nsp14_AIG44 (right panels) cells were left untreated (uninduced) or cultured for 48 h in the presence of 2.5 μg/ml DOX (induced). Whole-cell extracts prepared from each culture were subjected to Western blotting using the indicated antibodies. (**C**) 293F_Nsp14_wt2 (the upper two panels) and 293F_Nsp14_AIG44 (the lower panels) cells were left untreated (the upper most panels: uninduced) or cultured for 48 h in the presence of 2.5 μg/ml DOX (the lower two panels: induced). The cells were fixed and subjected to FISH using a Cy3-labeled oligo-dT_50_ probe. The cell nuclei were stained with Hoechst 33342. The cells were observed by confocal microscopy. Maximum-intensity projections of a single stack (10 consecutive slices, 0.35 μm z-distance) of images are shown. (**D**) 293F_Nsp14_wt2 (left panels) and 293F_Nsp14_AIG44 (right panels) cells were cultured for 48 h in the presence of 2.5 μg/ml DOX. The cells were fixed and subjected to IFA using the indicated antibodies. The cell nuclei were stained with Hoechst 33342. The cells were observed by confocal microscopy. In the merged pictures, the fluorescent signals of THOC2 and SC35 were pseudocolored red and green, respectively.

To analyze the genome-wide gene expression changes induced by Nsp14, doxycycline (DOX)-inducible cell lines were established (Figure 3A). As expected, the expression of the wild-type and D^331^→A mutant Nsp14 proteins was tightly regulated, and the GFP-fusion proteins were detectable only after DOX induction (Figure 3B and C). Expression of wild-type Nsp14, but not the D^331^→A mutant, induced nuclear accumulation of bulk poly(A)^+^ RNAs (Figure 3C), as observed in transiently expressed cells. The dissolution/coalescence of nuclear speckles, which is reminiscent of the phenomena observed upon splicing inhibition (74), also occurred in wild-type Nsp14-expressing cells (Figure 3D). We also noted that the cells underwent morphological changes upon the expression of wild type Nsp14, and most of the cells rounded up after 48 hr of DOX induction (Figure 3C, middle panels and Figure 3D, left panels).

**Figure 4.**
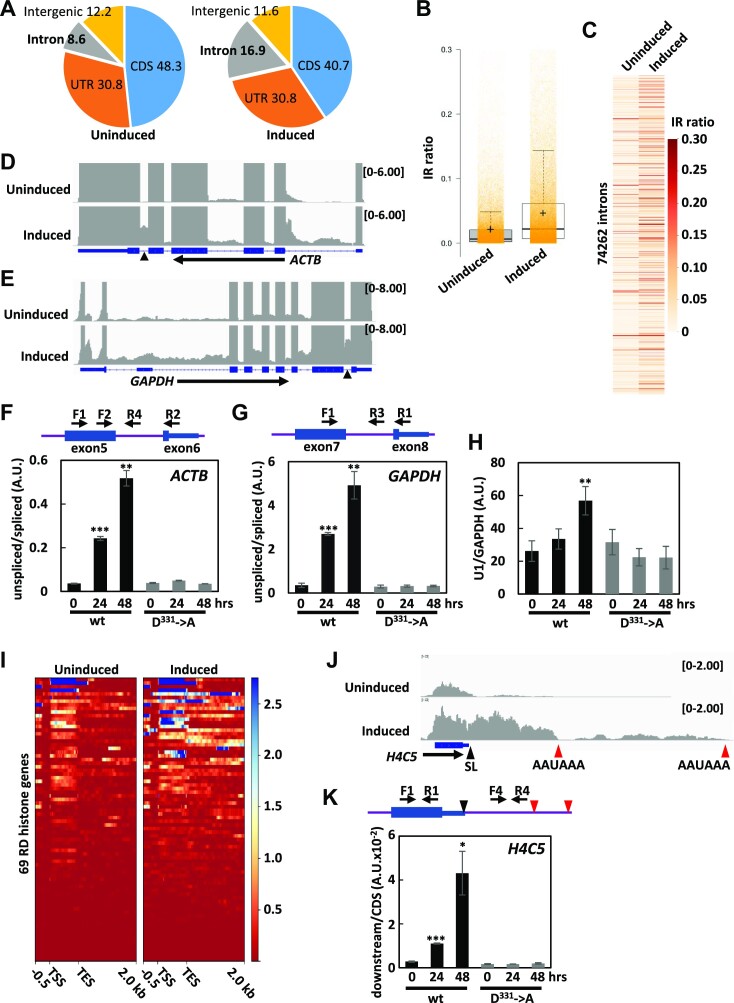
Gene expression analysis of the Nsp14-expressing cell line. Total RNA prepared from 293F_Nsp14_wt2 cells before (uninduced) or 48 h after (induced) DOX induction was subjected to poly(A)^+^-RNA-seq analysis. After adaptor trimming and quality filtering of the 101 base paired-end raw reads, the passed reads were mapped to the human genome 19 (hg19) assembly. (**A**) Distribution of the 4.89 × 10^9^ (uninduced) and 4.15 × 10^9^ (induced) mapped bases on different gene features was analyzed by CollectRNAseqMetrics from gatk4-4.1.6.0–0 and shown as percentages. CDS: coding sequence, UTR: 5′- and 3′-untranslated regions. (**B**, **C**) Intron retention ratios were calculated by IR finder. Overall changes in IR ratios (B) or changes at individual introns (C) are shown. In (B), the centerlines show the medians; the box limits indicate the 25th and 75th percentiles as determined by R software; the whiskers extend 1.5 times the interquartile range from the 25th and 75th percentiles; and the crosses show the means. Orange transparent dots are individual data points. (**D**, **E**) IGV view of the RNA-seq data at the *ACTB* and *GAPDH* loci. CPM-normalized RNA-seq read coverage of uninduced and induced samples is shown. (**F–H**) Total RNA prepared from 293F_Nsp14_wt2 and 293F_Nsp14_AIG44 cells cultured for the indicated periods in the presence of DOX was subjected to qRT−PCR analysis. The schematics shown above the graphs (F and G) are the relative positions of the PCR primers. In (F) and (G), the amounts of intron RNA were normalized to that of corresponding exon RNA. In (H), the amount of U1 snRNA was normalized to that of GAPDH. The data are presented as the means ± SDs of three biological replicates. ** and *** indicate *P* values <0.01 and <0.001, respectively. (**I**) Changes in RNA-seq read coverage at replication-dependent (RD) histone genes before (uninduced) and after (induced) Nsp14 expression. The heatmaps range from 0.5 kb upstream of the transcription start site (TSS) to 2 kb downstream of the transcription termination site (TES) of 69 RD histone genes. (**J**) IGV view of the RNA-seq read coverage of uninduced and induced samples at the histone *H4C5* locus. CPM-normalized read coverage was calculated by DeepTools. Red vertical arrowheads indicate the positions of the polyadenylation signal, whereas the black arrowheads indicate the position of the stem−loop-dependent cleavage site. (**K**) Total RNA prepared from 293F_Nsp14_wt2 and 293F_Nsp14_AIG44 cells before (0 h) or 24 and 48 h after DOX induction was subjected to qRT-PCR analysis using the indicated PCR primers. The amount of downstream RNA was normalized to that of CDS RNA. The data are presented as the means ± SDs of three biological replicates. * and *** mean *P* value <0.05 and <0.001, respectively. The schematic shown above the graph indicates the relative positions of the PCR primers. Black and red vertical arrowheads show the positions of the SL sequence and the canonical polyadenylation signals, respectively.

Total RNA prepared before (uninduced) and 48 hr after the induction (induced) of wild-type Nsp14 expression was subjected to poly(A)^+^ RNA-seq analysis. Of approximately 50 million paired reads each prepared from the samples, most (92.09% of uninduced and 93.24% of induced samples, Supplementary Figure S4) were uniquely mapped to the human reference genome hg19. The mapping rate of the reads to different gene features was further analyzed, and the intron reads were considerably increased (8.6 to 16.9%) upon Nsp14 expression at the expense of CDS reads (48.3 to 40.7%) (Figure 4A). To examine whether splicing defects actually occur, the intron-retention (IR) ratio was calculated (75). As shown in Figure 4B and C, the IR ratio was significantly increased by Nsp14 expression. Closer inspection of individual genes revealed that intron coverage was actually increased at different genes (Figure 4D and E). The increase in intron coverage at the *ACTB* and *GAPDH* loci was confirmed by qRT-PCR analysis, and wild-type Nsp14, but not the D^331^→A mutant, caused these changes (Figure 4F and G). Interestingly, in the IR analysis, nearly 25% of the retained introns were appended with the ‘nonUniformIntronCover’ warning. In fact, at long first introns of a subset of genes, premature polyadenylation was detected, which was similar to that observed when U1 snRNA was inhibited by antisense morpholino (Supplementary Figure S5A, C, D, E). The data indicate that a process called ‘telescripting’ (55) was affected. Since the relative amount of U1 snRNA was increased (Figure 4H) and uncleaved pre-U1 snRNA was virtually undetectable under Nsp14-expressing conditions, the downregulation and/or malfunction of U1 snRNP *per se* was not the direct cause of this defect.

Of approximately 50,000 significantly expressed transcripts, most were downregulated, and only 25% were upregulated upon Nsp14 expression. Of note, the poly(A)^+^ RNA-seq read coverage on a subset of the RD histone genes was increased upon Nsp14 expression (Supplementary Table S4). Normally, transcription of the RD histone genes is terminated just downstream of the stem−loop (SL) sequence by SLBP-dependent cleavage, and therefore, the RD histone mRNAs are nonadenylated (76,77). Nsp14 inhibited SLBP-dependent RD histone termination, and the cleavage sites shifted toward the downstream canonical polyadenylation sites (Figure 4I and J). As the result, the amount of polyadenylated RD histone mRNAs was increased significantly. An increase in the RNA level downstream of the SL sequence of the *H4C5* gene was confirmed by qRT−PCR analysis (Figure 4K). Termination by the canonical cleavage/polyadenylation factors at other genes with alternative polyadenylation sites, such as the *TIMP2* and *RPL22* genes, was not grossly altered (see Supplementary Figure S6), indicating that the downstream shift of the polyadenylation site is specific for the RD histone genes.

### Nsp14 increases the cellular concentration of m7GTP

**Figure 5.**
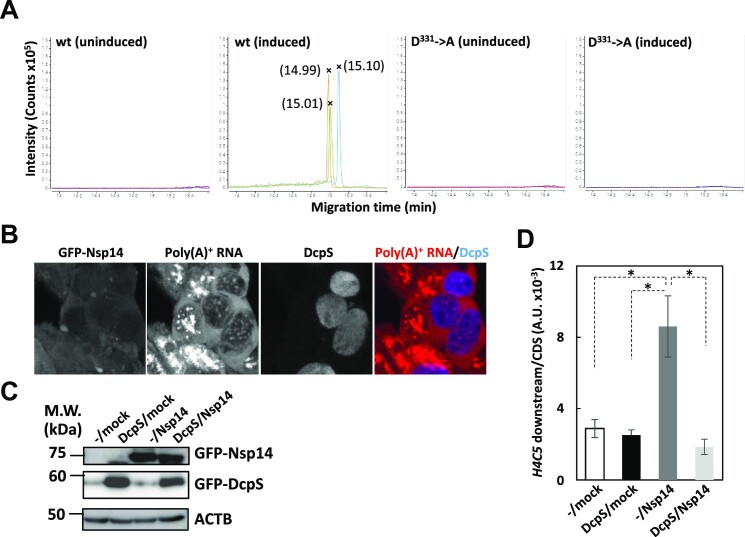
Production of m7GTP in Nsp14-expressing cells. (**A**) Detection of m7GTP in extracts prepared from cells cultured under the indicated conditions using CE-MS. Each analysis was performed in triplicate. Peak migration times are shown in parentheses. (**B**) 293F_Nsp14_wt2 cells were transiently transfected with the FLAG-tagged DcpS expression vector. Twenty-four hours after transfection, 2.5 μg/ml DOX was added to the culture medium, and the cells were cultured for an additional 48 h. The cells were fixed and subjected to IFA using anti-FLAG M2 mAb followed by FISH using a Cy3-labeled oligo-dT_50_ probe. The cells were observed by confocal microscopy. Maximum intensity projections of a single stack (10 consecutive slices, 0.35 μm *z*-distance) of images are shown. In the merged picture, the fluorescent signals of FLAG-DcpS and poly(A)^+^ RNAs were pseudocolored blue and red, respectively. (**C**, **D**) 293F_hACE2_DcpS_29 cells were left untreated (–) or induced by DOX for 24 h (DcpS). The cells were mock transfected (mock) or transfected with the GFP-Nsp14 expression vector (Nsp14) and cultured for an additional 48 h. (C) Whole-cell extracts were subjected to Western blotting using the indicated antibodies. (D) Total RNA was subjected to qRT-PCR analysis as in Figure 4K. The amount of downstream RNA was normalized to that of CDS RNA. The data are presented as the means ± SDs of three biological replicates. * means *P* value < 0.05.

SARS-CoV Nsp14 reportedly methylates not only the cap guanosine, but also the guanosine base of ‘free’ mononucleotide GTP in *in vitro* methylation assays (69). Thus, we addressed whether the closely related SARS-CoV-2 Nsp14 actually increases the cellular m7GTP level. Cell extracts prepared before and after 48 h of DOX induction were subjected to capillary electrophoresis coupled with mass spectrometry (CE-MS). Strikingly, CE peaks with migration times of approximately 15 min were detected only in the extracts prepared from wt Nsp14 expressing cells (Figure 5A). When the extracts prepared either from uninduced cells or from cells expressing the D331→A mutant were analyzed, nothing was detected in the same migration time range (Figure 5A). The average monoisotopic m/z of the CE peaks was 267.4987, which was almost identical to the theoretical m/z of m7GTP bivalent cation (267.4959), indicating that Nsp14 actually methylates GTP. Moreover, the results indicate that the D331→A mutant is enzymatically inactive. The slight difference in the migration times between the samples and synthetic m7GTP was most likely due to the presence of residual ingredients originating from the culture medium or cell extracts, because the peak was indistinguishable from the spike-in m7GTP standard (Supplementary Figure S7).

The scavenger mRNA decapping enzyme DcpS (78) eliminates cellular m7GTP by converting it to N7-methylated GMP (79). When DcpS was overexpressed in Nsp14-expressing cells, the poly(A)^+^ RNA export block was considerably mitigated and the size and the shape of the nuclear speckles returned to normal (Figure 5B). In addition, the increased readthrough *H4C5* RNA level by Nsp14 was significantly reduced by the DOX-induced expression of DcpS, although DcpS expression alone did not significantly alter it (Figure 5C and D, see also Supplementary Figure S9D for establishment of the cell line). From these data, we concluded that SARS-CoV-2 Nsp14 produces m7GTP *in vivo* and that the accumulated m7GTP inhibited mRNA nuclear export and RD histone mRNA 3′-end formation.

### Nsp14 inhibits the NCBC-cap interaction

**Figure 6.**
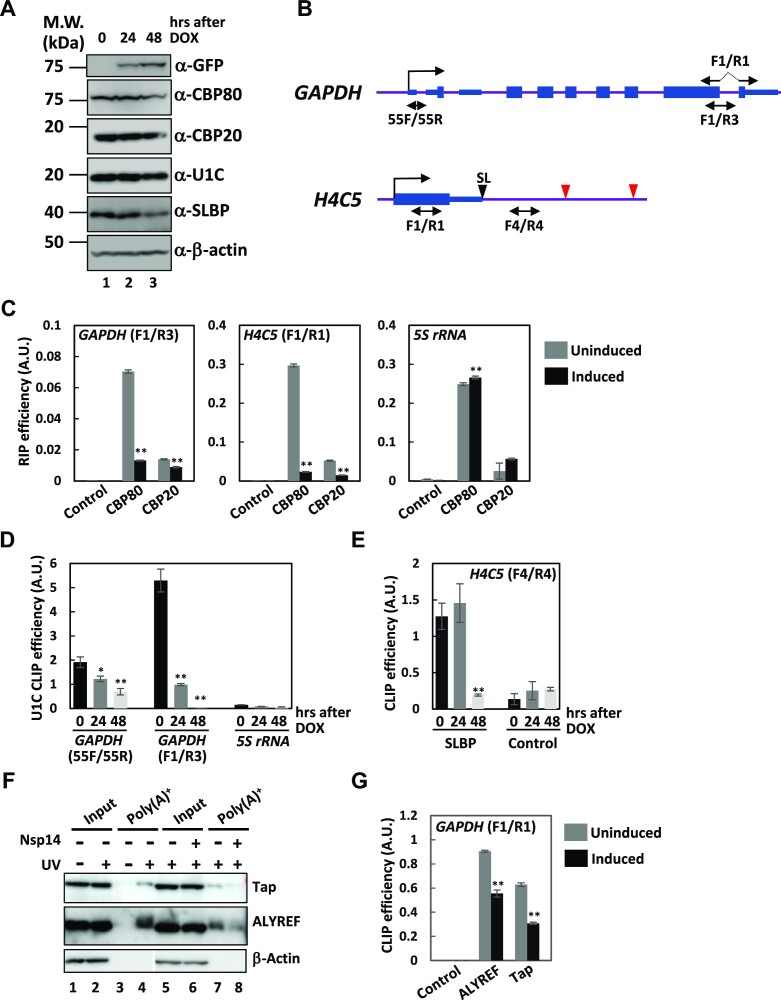
Impairment of mRNA recognition by NCBC, SLBP, and mRNA export factors in Nsp14-expressing cells. (**A**) Extracts prepared from 293F_Nsp14_wt2 cells before (0 h), 24 and 48 h after DOX induction were subjected to Western blotting using the indicated antibodies. The positions of molecular weight markers are indicated on the left in kilodaltons. (**B**) Structures of the *GAPDH* and *H4C5* loci. Thin and thick boxes indicate UTRs and CDSs, respectively, whereas the lines indicate introns or intergenic regions. Vertical lines with horizontal arrows indicate (major) transcription start sites. The positions of the PCR amplicons are shown below the schematics. The positions of the PCR primers used for amplification of the spliced *GAPDH* mRNA (F1 and R1) are shown above the schematics. Black and red arrowheads indicate the positions of the AAUAAA hexamer. Note that the schematics are not drawn to scale. (**C**) RIP analysis was carried out before (uninduced: gray) and 48 h after (induced: black) the induction of Nsp14. Input RNAs and RNAs copurified with anti-CBP80 and anti-CBP20 antibodies as well as control antibodies were subjected to qRT−PCR with the indicated PCR primers. RIP efficiency was calculated by dividing the amounts of RNAs immunopurified with each antibody by that of the corresponding input. The data are presented as the means ± SDs of three technical replicates. ** means *P* value < 0.01. (**D**) Whole-cell extracts prepared from UV-crosslinked 293F_Nsp14_wt2 cells induced for the indicated periods were immunoprecipitated with an anti-snRNP U1C antibody. The amounts of *GAPDH* pre-mRNA and 5S rRNA in the immune-pellets were divided by that in the corresponding input to calculate CLIP efficiency. Shown is a representative of three independent experiments. The data are presented as the means ± SDs of three technical replicates. * and ** indicate *P* values < 0.05 and < 0.01, respectively. (**E**) Whole-cell extracts prepared from UV-crosslinked 293F_Nsp14_wt2 cells induced for the indicated periods were immunoprecipitated with an anti-SLBP antibody. The amounts of *H4C5* pre-mRNA in the immune pellets were divided by the amounts in the corresponding input to calculate CLIP efficiency. Shown is a representative of three independent experiments. The data are presented as the means ± SDs of three technical replicates. ** means *P* value < 0.01. (**F**) The parental 293F (lanes 1–4) and 293F_Nsp14_wt2 (lanes 5–8) cells treated as indicated were subjected to UV crosslinking. The UV dose was 300 mJ/cm^2^. Expression of Nsp14 was induced for 48 h. Poly(A)^+^ RNPs (Poly(A)^+^: lanes 3, 4, 7, 8) were purified from whole-cell extracts (input: lanes 1, 2, 5, 6) prepared from each cell culture. The samples were analyzed by Western blotting using the antibodies indicated on the right of each panel. The lowest panel is a composite of relevant areas of two separate membranes. (**G**) Whole-cell extracts prepared from UV-crosslinked 293F_Nsp14_wt2 cells with (induced) or without (uninduced) Nsp14 expression were immunoprecipitated with anti-ALYREF and anti-Tap as well as control antibodies. The amount of *GAPDH* mRNA in the immune pellet was divided by that in the corresponding input to calculate CLIP efficiency. Shown is a representative of two independent experiments. The data are presented as the means ± SDs of three technical replicates. ** means *P* value < 0.01.

The data thus far indicate that m7GTP produced by Nsp14 caused the observed defects in gene expression; i.e. pre-mRNA splicing, histone mRNA 3′-end formation, and mRNA nuclear export. We conceived that these seemingly divergent outcomes could converge to functional defects of NCBC. As the mRNA cap and NCBC interaction can be competitively inhibited by m7GTP (13), we first examined whether the interaction between the capped RNAs and the components of NCBC is affected by Nsp14. To this end, RNA immunoprecipitation (RIP) using anti-CBP80 and -CBP20 antibodies was carried out. As determined by Western blotting, the expression of CBP80 and CBP20 was not changed significantly upon Nsp14 expression (Figure 6A, also see Supplementary Figure S8 for the expression and localization of CBP20 in individual cells). The amounts of capped RNAs coprecipitated with either CBP80 or CBP20 were severely decreased after Nsp14 induction (Figure 6C, see Figure 6B for primer positions). In contrast, the background precipitation of uncapped 5S rRNA with the NCBC components was unaffected or slightly increased upon expression of Nsp14 (Figure 6C). These data indicate that cap recognition by NCBC is abated by Nsp14 expression.

### Nsp14 impairs both U1 snRNP and SLBP recruitment to pre-mRNAs

Given the absence of functional NCBC on nascent pre-mRNAs, we hypothesized that the subsequent recruitment of pre-mRNA processing factors might also be affected by Nsp14. To examine whether Nsp14 alters the recruitment of U1 snRNP and SLBP to pre-mRNAs, CLIP followed by qRT−PCR was carried out by using antibodies specific for U1C and SLBP. Western blotting using these antibodies indicated that the expression of U1C was mostly unaffected (∼80%) and SLBP was decreased (∼40%) at 48 h after Nsp14 induction (Figure 6A). Coprecipitation of *GAPDH* pre-mRNA with anti-U1C antibody was substantially decreased upon Nsp14 expression (Figure 6D, 55F/55R and F1/R3). As the anchoring of U1 snRNP to long first introns via NCBC with Ars2 has been implicated in telescripting (55), coprecipitation of the *ACTN4* pre-mRNA with anti-U1C was also severely impaired by Nsp14 (Supplementary Figure S5B). Coprecipitation of 5S rRNA was almost undetectable (Figure 6D), indicating the specificity of our experimental settings. In addition, the interaction of SLBP with RD histone *H4C5* pre-mRNA was also decreased to background levels (Figure 6E). The extent of the reduction in RNA coprecipitation with either U1C or SLBP far exceeded that at the protein level. Therefore, from these data, we concluded that Nsp14 disturbs the recruitment of U1 snRNP and SLBP to pre-mRNAs.

### Nsp14 inhibits the formation of export-competent mRNP

To elucidate the molecular mechanism of the defect in mRNA nuclear export caused by Nsp14, association of transport factors with poly(A)^+^ RNAs was examined by UV-crosslinking. As shown in Figure 6F, only upon UV-irradiation were both Tap and ALYREF recovered in the poly(A)^+^ RNA-containing fraction (Figure 6F, lanes 1–4). The crosslinking of Tap and ALYREF to poly(A)^+^ RNAs was severely impaired under Nsp14-expressing conditions (Figure 6F, lanes 5–8). The association of these mRNA export factors with *GAPDH* mRNA was further examined by CLIP followed by qRT-PCR. The amount of *GAPDH* mRNA associated with ALYREF and Tap was significantly reduced (Figure 6G). These data indicate that Nsp14 impairs host mRNA export by inhibiting the recruitment of export factors.

### Gene expression changes in SARS-CoV-2-infected cells

**Figure 7.**
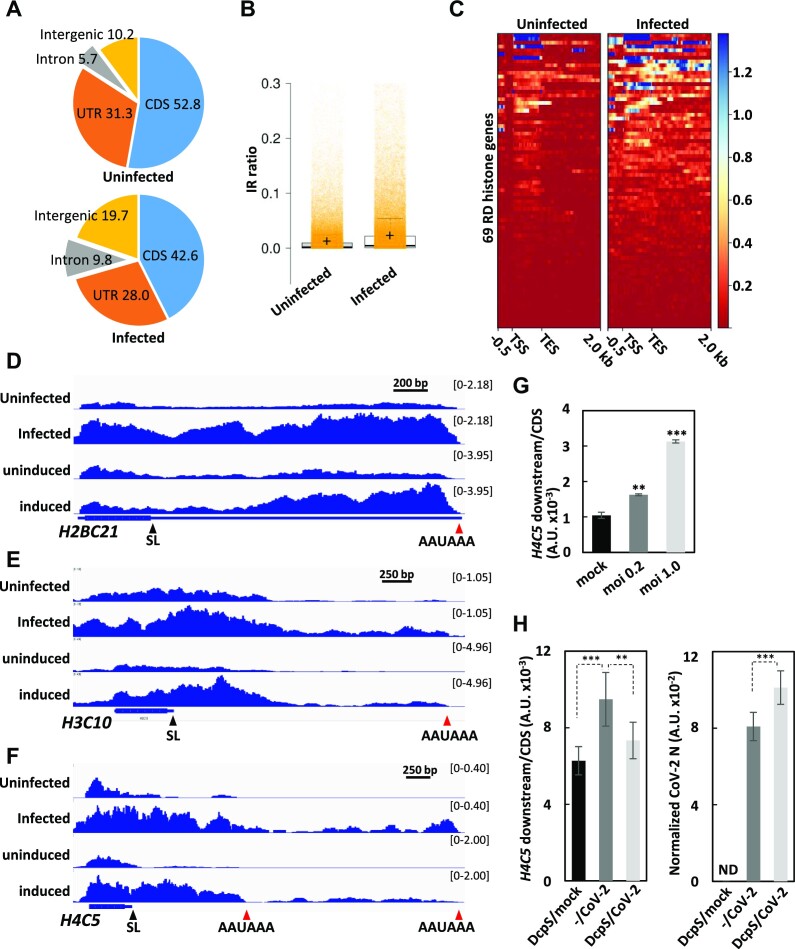
Gene expression analysis of SARS-CoV-2-infected cells. (**A**) Distribution of the 9.48 × 10^9^ (uninfected) and 8.49 × 10^9^ (infected) mapped bases on different gene features was analyzed by CollectRNAseqMetrics from gatk4-4.1.6.0–0 and shown in percentages. CDS: coding sequence, UTR: 5′- and 3′-untranslated regions. (**B**) Intron retention ratios were calculated by IR finder. Overall changes in IR ratios are shown. The centerlines show the medians; the box limits indicate the 25th and 75th percentiles as determined by R software; the whiskers extend 1.5 times the interquartile range from the 25th and 75th percentiles; and the crosses show the means. Orange transparent dots are individual data points. (**C**) Changes in RNA-seq read coverage at replication-dependent (RD) histone genes before (uninfected) and after (infected) SARS-CoV-2 infection. The heatmaps range from 0.5 kb upstream of the transcription start site (TSS) to 2 kb downstream of the transcription termination site (TES) of 69 RD histone genes. (**D**–**F**) IGV view of the RNA-seq data at the *H2BC12*, *H3C10* and *H4C5* loci. The CPM-normalized RNA-seq read coverage of uninfected and infected samples is shown (top two rows). As a comparison, the read coverage of control (uninduced) and Nsp14-expressing (induced) samples is also shown (bottom two rows). (**G**) Total RNA prepared from 293F_hACE2_21 cells infected with SARS-CoV-2 at the indicated multiplicity of infection (MOI) was subjected to qRT-PCR analysis as in Figure 4K. The amount of the downstream RNA was normalized to that of the CDS RNA. Shown is a representative of two independent experiments. The data are presented as the means ± SDs of three technical replicates. ** and *** indicate *P* values <0.01 and <0.001, respectively. (**H**) 293F_hACE2_DcpS_29 cells were left untreated (–) or induced GFP-DcpS expression by DOX for 24 h (DcpS). The cells were infected with SARS-CoV-2 at an MOI of 1.0 and cultured for another 24 h. *Left*: Total RNAs prepared from each culture were subjected to qRT-PCR to analyze the read-through transcription of the *H4C5* locus as in Figure 4K. The data are presented as the means ± SDs of three biological replicates. ** and *** mean *P* values <0.01 and <0.001, respectively. *Right*: The amount of SARS-CoV-2 N mRNA normalized to that of *GAPDH* mRNA was also quantitated. The data are presented as the means ± SDs of three biological replicates. *** means *P* value <0.001. ND means none detected.

Finally, to assess the gene expression changes in SARS-CoV-2-infected cells, we analyzed publicly available poly(A)^+^ RNAseq data (54) (see Supplementary Figure S4 for mapping details). Intronic reads were increased significantly by SARS-CoV-2 infection, whereas CDS reads were decreased (Figure 7A). As shown in Figure 7B, the IR ratio was also increased genome-wide in infected cells. In addition, increases in both the readthrough transcript and polyadenylated mRNA levels of RD histone genes were observed, indicating that SLBP-dependent 3′-end formation was perturbed (Figure 7C–F). In this analysis, we noted that intergenic reads were also increased (Figure 7A), which was not obvious in Nsp14-expressing cells (Figure 4A). This might be due to the presence of other viral factors. Although less pronounced than that in Nsp14-expressing cells, we confirmed the increase in the readthrough transcript level of the *H4C5* locus in SARS-CoV-2-infected 293F_hACE2 cells (Supplementary Figure S9) by qRT−PCR analysis (Figure 7G). Importantly, the defect in RD histone mRNA 3′-end processing by SARS-CoV-2 infection was alleviated by DcpS expression (Figure 7H, left). DcpS expression itself seemed not to decrease SARS-CoV-2 infectivity, as judged by viral N gene expression (Figure 7H, right). Thus, these data indicate that gene expression changes similar to those observed in Nsp14 expressing cells actually occur in SARS-CoV-2 infected cells.

## DISCUSSION

SARS-CoV-2 disrupts the host gene expression system in various means to escape from antiviral defenses. The disturbance of gene expression also serves as a basis for diverse pathologies associated with SARS-CoV-2 infection. Although SARS-CoV-2 completes its life cycle exclusively in the cytoplasm, different steps in the host gene expression process in both the nucleus and the cytoplasm are influenced by different viral factors (41,80). In this study, we have successfully identified Nsp14 as a novel viral mRNA export inhibitor. On analysis, the inhibitory effects were found not to be limited to mRNA export but extended to the nuclear mRNP maturation processes, all of which are accountable for NCBC dysfunction.

### m7GTP as a mediator of host gene expression shutoff by Nsp14

Among the different SARS-CoV-2 factors involved in host gene expression shutdown mechanisms, Nsp14 has been linked to the inhibition of transcription, splicing and translation (81,82). However, elucidation of the detailed molecular basis of the inhibition by this viral factor has been hampered mostly due to the absence of experimental evidence of m7GTP production in cells.

Despite the distinctive structural features (83), the N7-MTase activity of Nsp14 of SARS-CoV, a closely related species of SARS-CoV-2, is essentially the same as that of the cellular counterpart (84). Indeed, SARS-CoV Nsp14 is able to complement the growth of the otherwise lethal yeast *abd1* mutant (85). One notable difference between the human and viral N7-MTases is their substrate specificity, and SARS-CoV Nsp14 has been shown to methylate GTP in *in vitro* assays (69,86). In this study, we extended the previous *in vitro* data and unequivocally identified m7GTP produced in cells expressing SARS-CoV-2 Nsp14. As has been predicted from circumstantial evidence, our data have established that the inhibition of mRNA cap function by m7GTP does indeed underlie the virulence of this viral protein. We have also identified additional and previously unappreciated abnormalities brought about by this viral factor: defects in bulk poly(A)^+^ RNA export, the suppression of premature transcription termination (telescripting) and the 3′-end formation of RD histone mRNAs, all of which are also attributable to the inhibition of mRNA cap function (15). Intriguingly, most of these changes were also detectable in SARS-CoV-2-infected cells. The inhibition of host gene expression via the production of the small molecule m7GTP described in this report is an unprecedented strategy among other viral factors.

### The mechanism of the inhibition

The cap structure m7GpppN (where N is any nucleotide) is a characteristic feature of all eukaryotic mRNAs. The cap is important not only for protecting mRNAs from exonucleolytic degradation but also for providing a binding site to NCBC and eIF4E, which play pivotal roles in nuclear mRNP maturation and cytoplasmic translation initiation, respectively (15,87). Thus, excess cap analogs impede eukaryotic gene expression at different levels by limiting the available cap binding proteins from mRNAs. Our data clearly indicate that Nsp14, via the production of m7GTP, disturbs cap recognition by NCBC and the subsequent recruitment of mRNA processing and export factors.

Each cap binding protein exhibits different affinities to different cap analogs. For example, eIF4E prefers capped short ribonucleotides (m7GpppG + N_20_: *K*_D_ ∼3 nM) to either m7GTP or m7GpppG (*K*_D_ ∼260 nM). In contrast, NCBC binds m7GTP and m7GpppN with much higher affinities (*K*_D_ ∼10 nM and ∼100 pM, respectively) (87). The rough estimate of m7GTP concentration in the Nsp14-expressing cells deduced from our CE-MS data was > 100 μM, which far exceeds the *K*_D_ values of the cap binding proteins to the methylated mononucleotide. In addition, cells express similar numbers of CBP20 (2.59 × 10^4^ /cell) and eIF4E (1.8 × 10^4^/cell) molecules (88). Hence, it is conceivable that under such extremely high m7GTP condition, the direct and simultaneous inhibition of both NCBC and eIF4E would be possible. In an *in vitro* reticulocyte lysate system, 100 μM of m7GTP is already high enough to efficiently inhibit translation (89). We also observed reduced incorporation of puromycin to nascent polypeptides in Nsp14-expressing cells (Supplementary Figure S10), indicating that translation is actually inhibited in our experimental condition. However, in a situation where only a limited amount of m7GTP is produced, NCBP would be preferable and a principal target. Consequently, nuclear phenotypes might be dominant over translational disturbance.

The scavenger decapping enzyme DcpS hydrolyzes both m7GpppN cap and m7GTP, which are intermediates of the 3→5 and 5→3 mRNA decay pathways, respectively, into metabolically ‘inert’ m7GMP (79). The hydrolyzing activity is therefore important for circumventing the competition between the residual cap and mRNAs as well as for preventing the misincorporation of methylated ribonucleotide into RNAs, both of which are potentially harmful for cells. In fact, the depletion of DcpS by shRNAs resulted in splicing defects (90), which coincide well with our observations in Nsp14-expressing cells and in SARS-CoV-2-infected cells. We also found that overexpression of DcpS counteracted Nsp14 and alleviated the defect in RD histone mRNA 3′-end formation as well as the block in bulk poly(A)^+^ RNA export, the regulation of which was proposed as a possible DcpS function more than a decade ago (91). Thus, another persuasive and non-mutually exclusive inhibitory mechanism is that m7GTP indirectly impedes mRNA cap function via suppression of the cap degradation process. Mammalian DcpS hydrolyzes m7GTP at a slower rate than m7GpppG (92). By slowing DcpS-mediated cap catabolism, Nsp14 could induce toxic accumulation of very short mRNA fragments with the cap structure, which perturbs the cap binding proteins more efficiently.

Surprisingly, our CE-MS analysis also revealed the downregulation of cellular ribonucleotides, especially their monophosphate forms, by Nsp14 (Supplementary Table S5). Interestingly in this context, Nsp14 appeared to upregulate *SMN2* gene expression (see Supplementary Table S4), which also occurred when DcpS was inhibited by small-molecule inhibitors, although the molecular mechanisms and biological significance of this phenomenon have yet to be addressed (93). That RNA catabolism is the major source of cellular CMP and UMP pools (94) and that there are functional links between the RNA degradation process and either NCBC or DcpS (78,95) suggest that these might be additional evidence that Nsp14 targets the cap catabolism.

### Implications for viral pathogenesis

Although several factors have already been attributed (41–44,46,80), the splicing and nuclear export defects caused by Nsp14 could contribute to those observed in SARS-CoV-2 infected cells. As discussed elsewhere (41–44,46,80), blocking gene expression at these levels might disturb invocation of the host defense system.

We also found that the recruitment of SLBP to RD histone mRNA via NCBC was severely impaired by Nsp14, resulting in the increased expression of polyadenylated RD histone mRNAs. SLBP is required not only for 3′-end formation but also for efficient nuclear export, cytoplasmic translation and stabilization of RD histone mRNAs (96). As a consequence, it is anticipated that the expression of the RD histone genes is distorted by Nsp14. It has also been reported that SLBP depletion causes S-phase arrest (97,98). During the course of this study, we noted that the expression of Nsp14 considerably delayed cell growth. We found that SLBP, which is synthesized immediately prior to entry into S-phase and degraded rapidly as cells exit from S-phase (77), was downregulated upon the expression of Nsp14. Moreover, the expression of RD histone and a subset of cell cycle regulators such as cyclin A, geminin, and Emi1, all of which increase as S-phase proceeds (99,100), was found to be reduced in Nsp14-expressing cells (Supplementary Figure S11A and B), suggesting that Nsp14 impairs cell cycle progression during S-phase. Thus, one possibility is that inefficient supply of RD histones by Nsp14 bolsters the recently reported cell cycle arrest during SARS-CoV-2 infection (101,102).

Neuronal impairment by direct SARS-CoV-2 infection of brain tissues is still under debate, and direct brain infection appears to be regarded as a rather uncommon mechanism (103). However, recent reports have established that SARS-CoV-2 is able to infect both astrocytes and neurons (104–106). NCBC has been linked to mRNP localization and local translation at neuronal processes, which are important for neuronal development and function (49). Intriguingly, loss-of-function mutations of *DCPS* cause inherited neuronal diseases, implicating DcpS, or more generally cap catabolism, in neuronal development and functions (107–109). Thus, another plausible scenario is that Nsp14, via its inhibition of NCBC functions, contributes to viral pathogenesis as a possible underpinning of the persistent cognitive symptoms associated with SARS-CoV-2 infection.

In summary, our present study uncovered the previously unrecognized relevance of m7GTP produced by SARS-CoV-2 Nsp14 to host gene expression shutdown mechanisms and diverse viral pathologies. Nsp14 would undoubtedly be a valuable *in vivo* tool to study the physiological and pathological functions of the mRNA cap and cap-binding proteins in more detail. In addition, our data suggest that the process of cap catabolism might be a novel target worth investigating for the development of treatments for the after-effects of SARS-CoV-2 infection, or long COVID.

## Supplementary Material

gkad483_Supplemental_Files

## Data Availability

RNA-seq data are available from the sequence read archive SRA under the accession numbers DRR440984 (uninduced sample) and DRR440985 (induced sample).
