# Supplementary material for: Nsp14 of SARS-CoV-2 inhibits mRNA processing and nuclear export by targeting the nuclear cap-binding complex
Source: Nucleic Acids Res. 2023 Jun 1;51(14):7602–18. doi: 10.1093/nar/gkad483 (PMC10415132; doi:10.1093/nar/gkad483)
Supplement: gkad483_Supplemental_Files [file gkad483_supplemental_files.zip › Katahira2023_Suppl_Final.pdf]

Supplementary data

Nsp14 of SARS-CoV-2 inhibits mRNA processing and nuclear export by targeting the nuclear cap binding complex

Jun Katahira, Tatsuya Ohmae, Mayo Yasugi, Ryosuke Sasaki, Yumi Itoh, Tomoko Kohda, Miki Hieda, Masami Yokota Hirai, Toru Okamoto, Yoichi Miyamoto

Supplementary figure legends

Figure S1. Cellular distribution of poly(A)$^{+}$ RNAs in SARS-CoV-2-infected cells. Vero E6 cells were infected with SARS-CoV-2. Twenty-four hours after infection, the cells were fixed and subjected to IFA using an anti-S antibody followed by FISH using a Cy3-labeled oligo-dT$_{50}$ probe. DNA was counterstained with Hoechst 33342. The cells were observed by confocal microscopy. In the merged picture, the fluorescent signals of DNA, viral S protein, and poly(A)$^{+}$ RNAs were pseudocolored blue, green, and red, respectively.

Figure S2. Distribution of poly(A)$^{+}$ RNAs in cells expressing individual viral proteins. HeLa cells were transfected with expression vectors harboring the indicated viral proteins. Forty-eight hours after transfection, the cells were fixed and subjected to IFA using an anti-Strep-tag II antibody followed by FISH using a Cy3-labeled oligo-dT$_{50}$ probe. DNA was counterstained with Hoechst 33342. The cells were observed by confocal microscopy.

Figure S3. Removal of GFP-tag does not affect the poly(A)$^+$ RNA export inhibitory activity of Nsp14. 293F cells were transiently transfected with the pCMV-Nsp14 vector. **(A)** Whole-cell extracts prepared from untransfected and the transfected cells were subjected to Western blotting using anti-Nsp14 antibody. **(B)** The transfected cells were fixed and subjected to IFA using anti-Nsp14 antibody followed by FISH using a Cy3-labeled oligo-dT$_{50}$ probe. The cells were observed by confocal microscopy. Maximum intensity projection of a single stack (10 consecutive slices, 0.35 μm z-distance) of images are shown. In the merged picture, the fluorescent signals of DNA, Nsp14, and poly(A)$^+$ RNAs were pseudocolored cyan, green, and red, respectively.

Figure S4. RNA-seq summary.

Figure S5. Nsp14 affects "telescripting". (A, C to E) IGV views of the RNA-seq read coverage at the *ACTN4* (A), *RAB7B* (C), *CSNK1G2* (D), and *AKAP13* (E) loci. The third and fourth rows show before (uninduced) and after (induced) Nsp14 induction, respectively. Red arrows indicate the positions of polyadenylation signals in the first introns. For comparison, the RNA-seq read coverage of control (SRR9864939) and anti-

U1 morpholino (SRR9864940)-treated cells are also indicated (first and second rows). **(B)** Whole-cell extracts prepared from UV-crosslinked 293F_Nsp14_wt2 cells induced for the indicated periods were immunoprecipitated with anti-snRNP U1C antibody. The amounts of *ACTN4* pre-mRNA in the immune-pellets were divided by that in the corresponding input to calculate CLIP efficiency. The data are presented as the means ± SDs of three technical replicates. ** means p value < 0.01. The approximate position of the qPCR amplicon (ACTN4 F1/R1) is shown in (A).

Figure S6. Nsp14 does not affect canonical polyadenylation. IGV views of the RNA-seq read coverage at the *TIMP2* and *RPL22* loci. The top and bottom rows in each panel show before (-) and after (+) Nsp14 induction, respectively. Vertical red arrow heads indicate approximate positions of the polyadenylation signal.

Figure S7. CE profile of the spike-in samples. The sample analyzed in Fig. 5A was mixed with H_2O (sample + H_2O) or m7GTP standard (sample + STD 100 μM) and subjected to CE analysis. Note that the standard peak is indistinguishable from that of the sample, indicating that the slight shift of the peaks is due to contaminants from the culture medium or cell lysates.

Figure S8. Expression and localization of CBP20 in Nsp14-expressing cells. 293F_Nsp14_wt2 cells were left untreated (upper panels) or subjected to DOX induction for 48 hr. The cells were fixed and subjected to IFA using an anti-CBP20 antibody. The cells were observed by confocal microscopy. In the merged pictures, the fluorescent signals of DNA, GFP-Nsp14, and CBP20 are pseudocolored blue, green, and red, respectively.

Figure S9. Establishment of a Rho-1D4 peptide-tagged hACE2-expressing 293F cell line. (A) Whole extracts prepared from the parental 293F and 293F_hACE2_21 cells were subjected to Western blotting using an anti-Rho-1D4 tag mAb. An anti-β-actin antibody was used to monitor loading. **(B)** The parental 293F and 293F_hACE2_21 cells were fixed and subjected to IFA using the anti-Rho-1D4 tag mAb. The nuclei were counterstained with Hoechst 33342. The cells were observed by confocal microscopy. The signals of DNA and hACE2 are pseudocolored cyan and green, respectively. **(C)** The cells indicated on the left were infected with SARS-CoV-2. Twenty-four hours after infection, the cells were fixed and subjected to IFA using the anti-S antibody. The cells were observed by phase-contrast and fluorescence microscopy. **(D)** 293F_hACE2_21

cells were transfected with linearized pInducer20-GFP-DcpS. Cells harboring the hACE2 and GFP-DcpS expression vectors were selected by 1.2 mg/ml G418 and 400 μg/ml hygromycin, and a clone designated 293F_hACE2_DcpS_29 was used for further analysis. DOX-inducible expression of the GFP fusion protein was confirmed by fluorescence microscopy. Note that the GFP-DcpS fusion protein is localized to the nucleus.

Figure S10. Translation inhibitory activity of Nsp14. 293F_Nsp14_wt2 cells were left untreated (lanes 1 to 4) or subjected to DOX induction for 48 hr (lanes 5 to 7). The cells were pulse-labeled with indicated concentrations of puromycin for 10 min and chased for an additional 60 min in drug-free medium. **(A)** Whole cell extracts prepared from each culture were subjected to SDS-PAGE followed by Western blotting using indicated antibodies (upper two panels) or Coomassie brilliant blue staining (bottom panel). **(B)** Quantification of the blots. Signal intensity of each lane of the anti-puromycin blot was divided by that of the corresponding anti-β-actin blot. The numbers below the graph correspond to the lane number.

Figure S11. Nsp14 reduced the expression of cell cycle genes and histone H4C5

mRNA. (A) Total RNA prepared from 293F_Nsp14_wt2 cells cultured for the indicated periods in the presence of DOX was subjected to qRT–PCR analysis. The amount of *H4C5* mRNA (amplified with the F1/R1 primer pair: see Fig. 4G) was normalized to that of *GAPDH* mRNA (amplified with the F1/R1 primer pair: see Fig. 4K). The data are presented as the means ± SDs of three biological replicates. * means p value < 0.05. **(B)** The whole-cell extracts used in Fig. 6 were reprobed with the indicated antibodies. The positions of molecular weight markers are indicated on the left in kilodaltons.

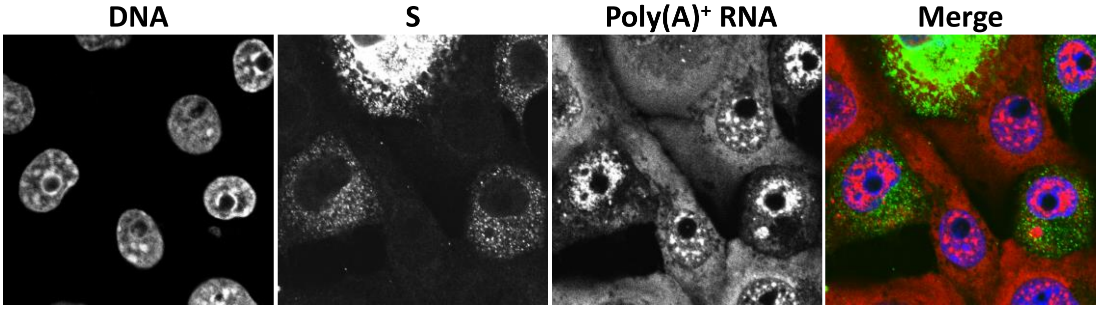
DNA
S
Poly(A)+ RNA
Merge

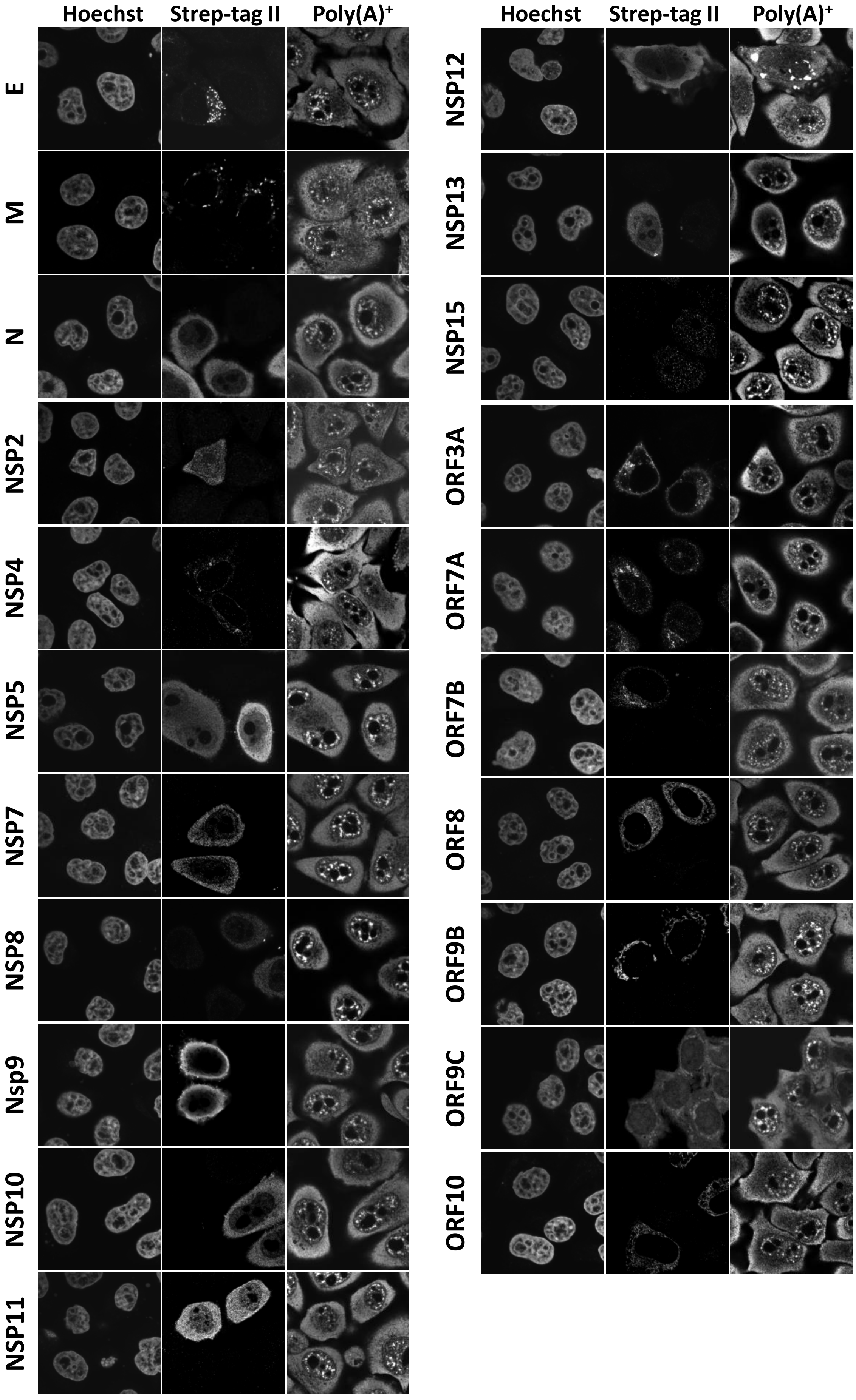

Hoechst
Strep-tag II
Poly(A)+
E
M
N
NSP2
NSP4
NSP5
NSP7
NSP8
Nsp9
NSP10
NSP11
Hoechst
Strep-tag II
Poly(A)+
NSP12
NSP13
NSP15
ORF3A
ORF7A
ORF7B
ORF8
ORF9B
ORF9C
ORF10

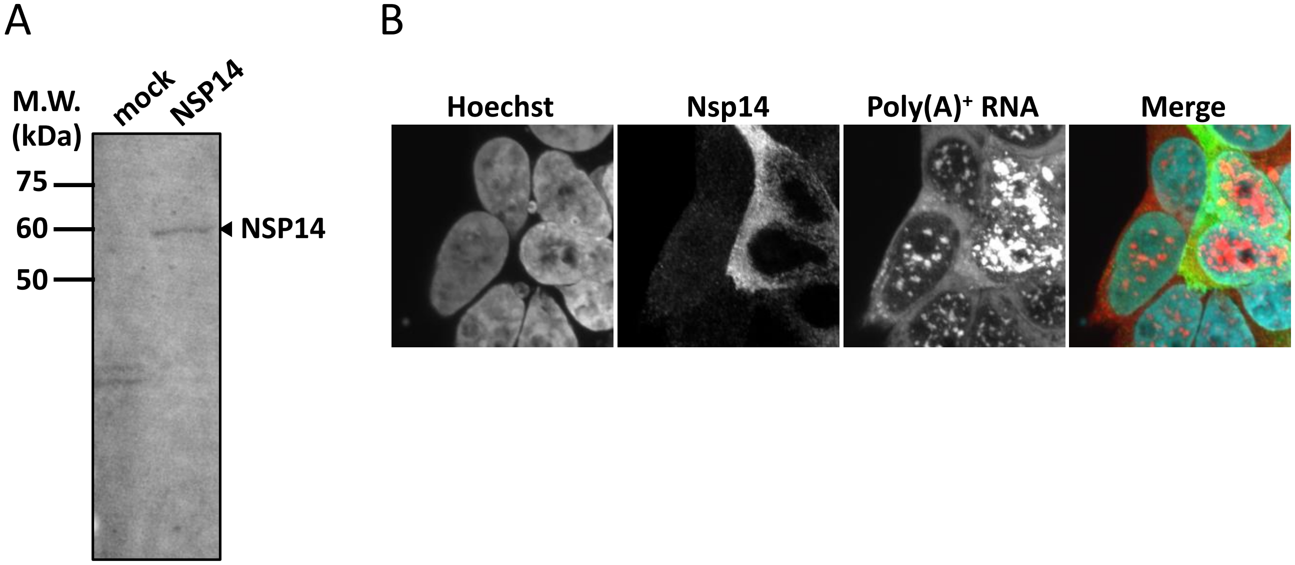
A
M.W.
(kDa)
mock
NSP14
75
60
50
NSP14
B
Hoechst
Nsp14
Poly(A)+ RNA
Merge

Nsp14 expression

	Uninduced	Induced
QC passed reads	24664002	21336218
Mapped reads	22713827	19894441
Mapped bases	4894111770	4149024727

SARS-CoV-2 infection

	Uninfected	Infected
QC passed reads	50238246	139806406
Mapped reads	46716176	42024114
Mapped bases	9475980305	8491978456

U1 antisense morpholino

	Control AMO	U1 AMO
QC passed reads	165824486	143164207
Mapped reads	143181949	121274451

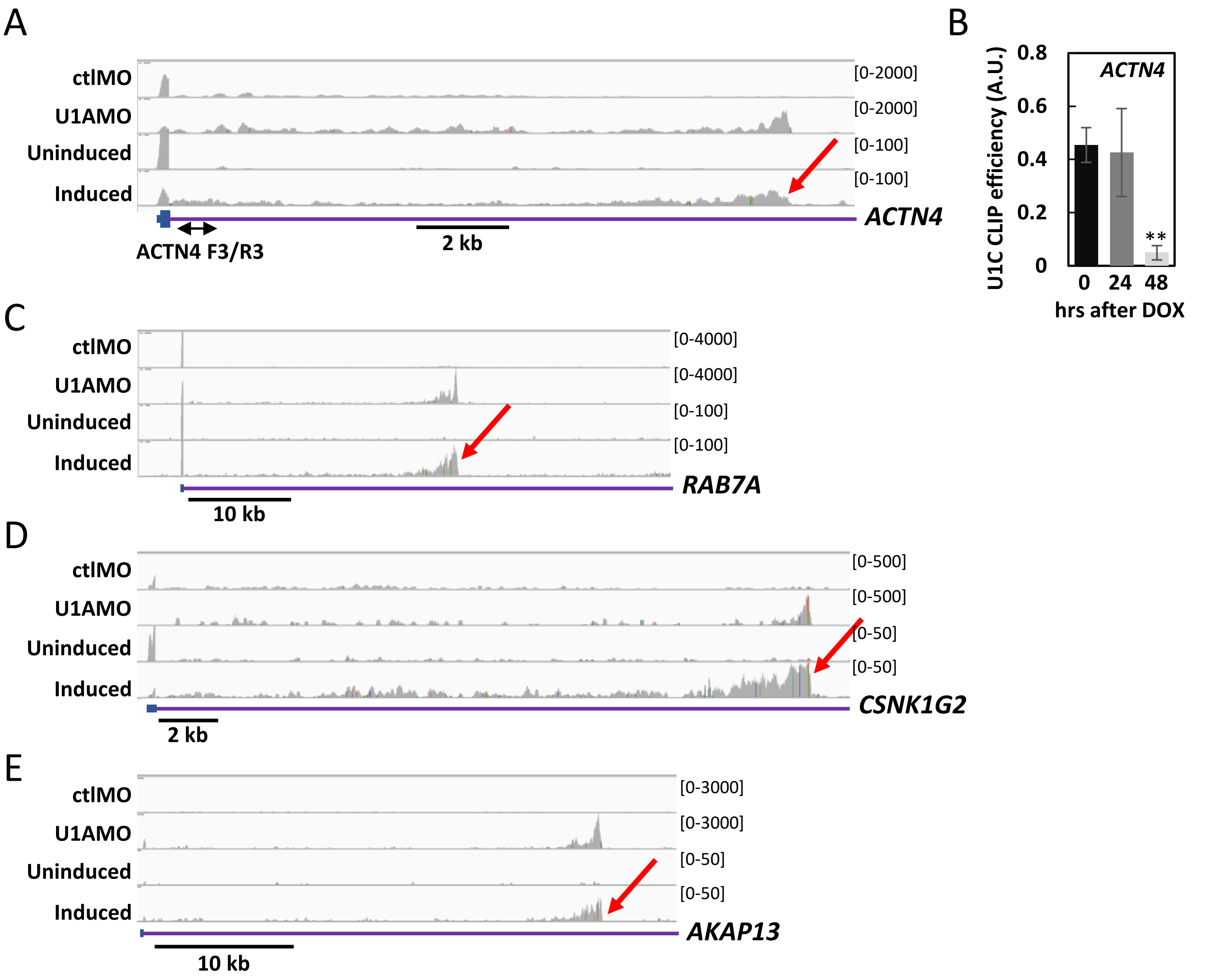
A
ctlMO
U1AMO
Uninduced
Induced
[0-2000]
[0-2000]
[0-100]
[0-100]
ACTN4
ACTN4 F3/R3
2 kb
B
ACTN4
U1C CLIP efficiency (A.U.)
0.8
0.6
0.4
0.2
0
**
0
24
48
hrs after DOX
C
ctlMO
U1AMO
Uninduced
Induced
[0-4000]
[0-4000]
[0-100]
[0-100]
RAB7A
10 kb
D
ctlMO
U1AMO
Uninduced
Induced
[0-500]
[0-500]
[0-50]
[0-50]
CSNK1G2
2 kb
E
ctlMO
U1AMO
Uninduced
Induced
[0-3000]
[0-3000]
[0-50]
[0-50]
AKAP13
10 kb

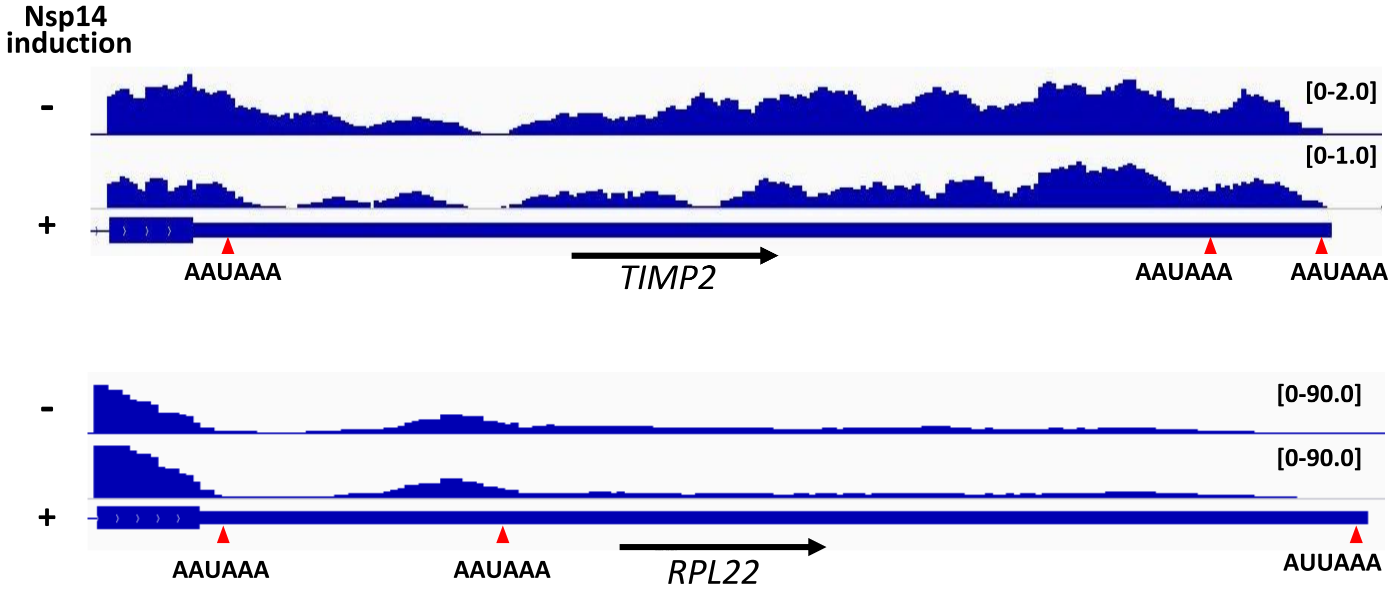
Nsp14
induction
-
[0-2.0]
[0-1.0]
+
AAUAAA
TIMP2
AAUAAA
AAUAAA
-
[0-90.0]
[0-90.0]
+
AAUAAA
AAUAAA
RPL22
AUUAAA

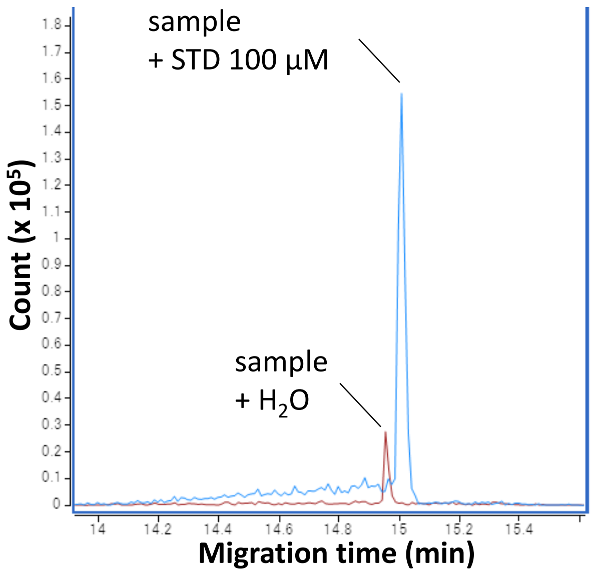
sample
+ STD 100 µM
sample
+ H2O
Count (x 10^5)
Migration time (min)
1.8
1.7
1.6
1.5
1.4
1.3
1.2
1.1
1
0.9
0.8
0.7
0.6
0.5
0.4
0.3
0.2
0.1
0
14
14.2
14.4
14.6
14.8
15
15.2
15.4

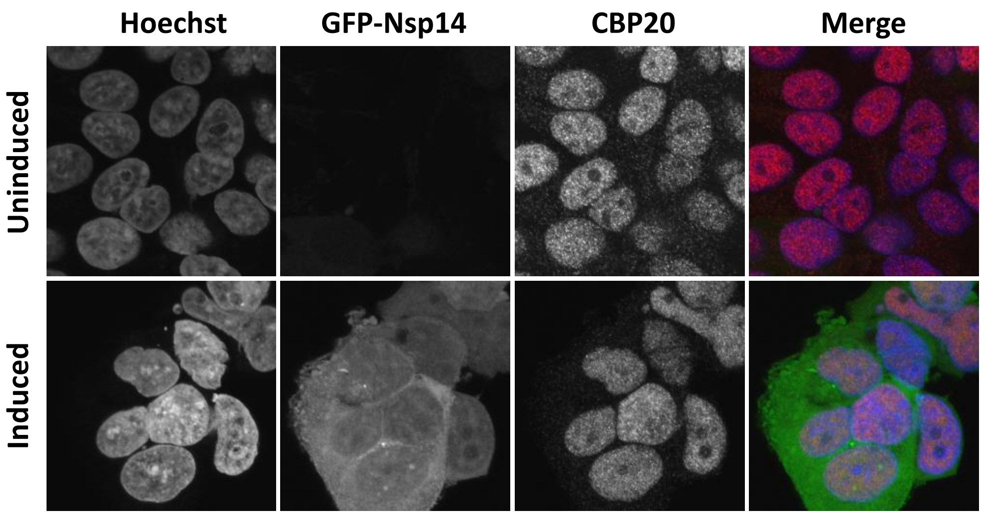
Hoechst
GFP-Nsp14
CBP20
Merge
Uninduced
Induced

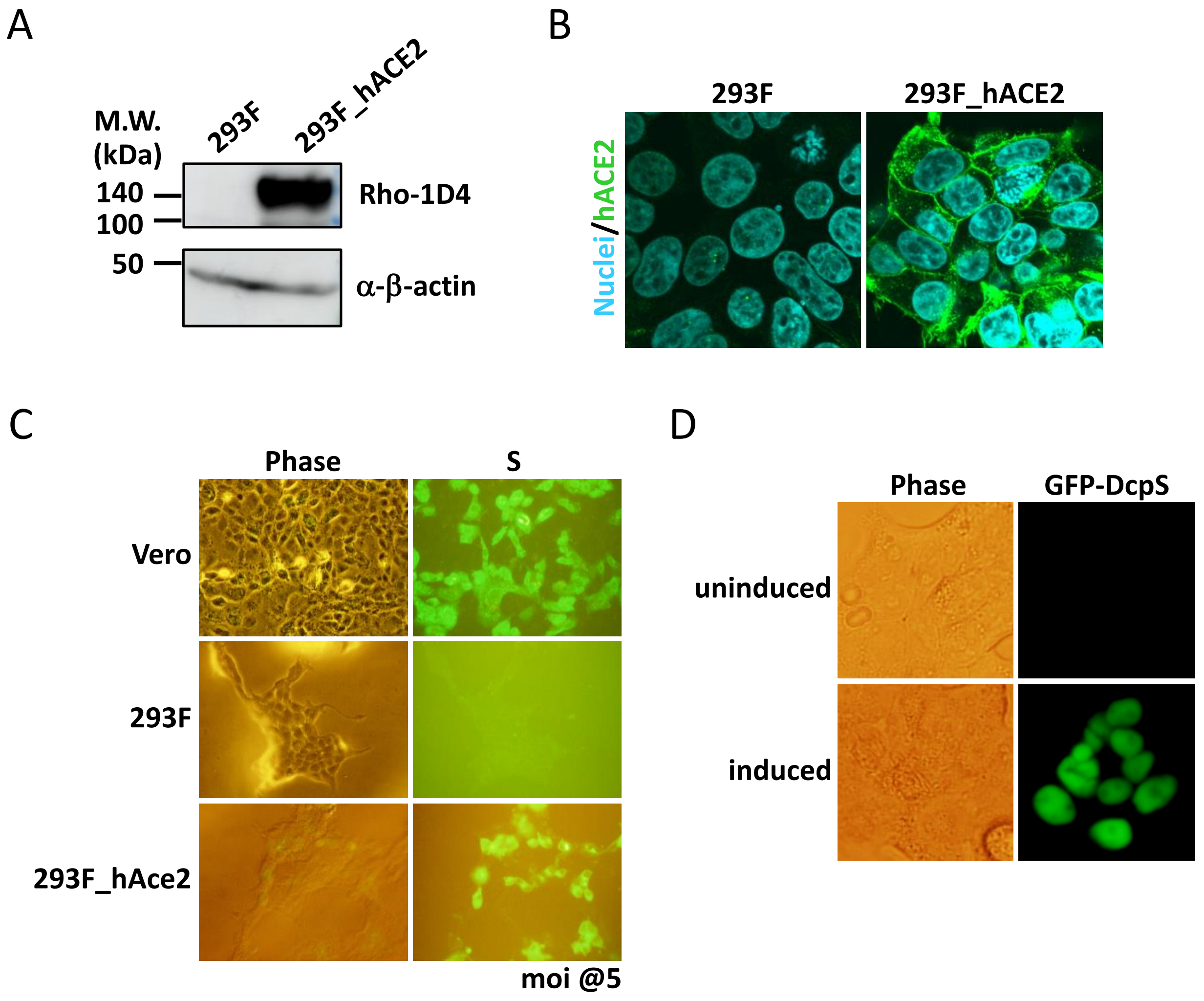
A
M.W. (kDa)
293F
293F_hACE2
140
100
50
Rho-1D4
α-β-actin
B
293F
293F_hACE2
Nuclei/hACE2
C
Phase
S
Vero
293F
293F_hAce2
moi @5
D
Phase
GFP-DcpS
uninduced
induced

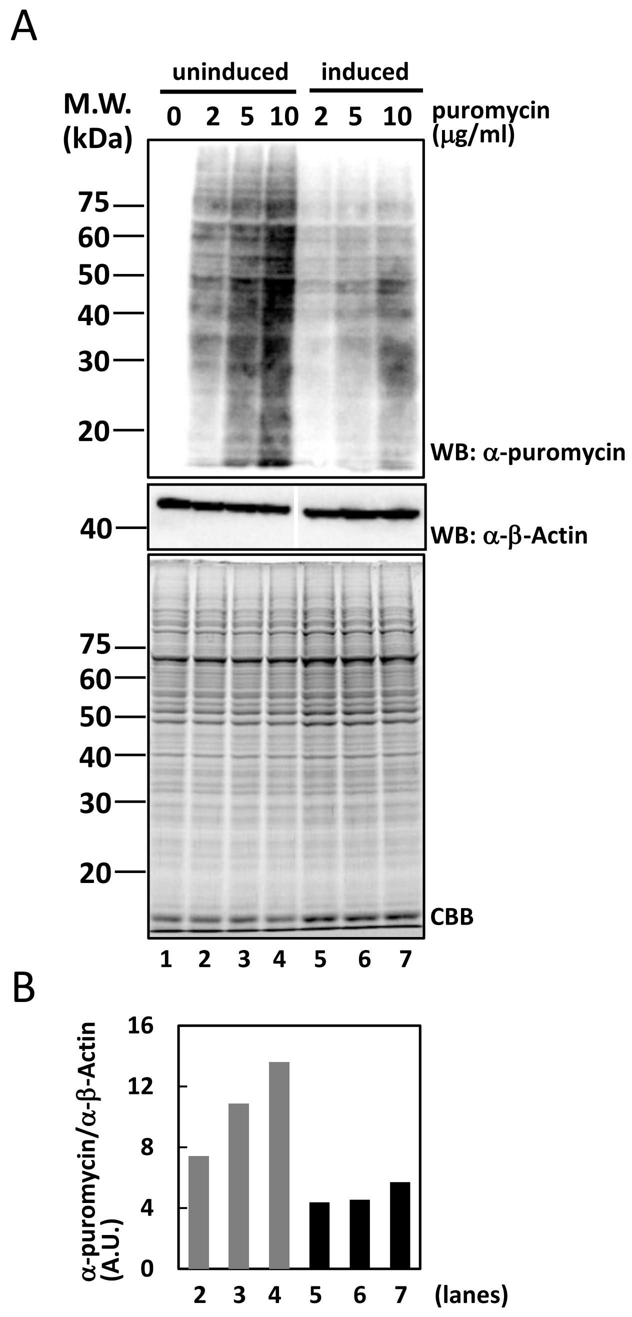
A
uninduced
induced
M.W.
(kDa)
0 2 5 10 2 5 10
puromycin
(μg/ml)
75
60
50
40
30
20
WB: α-puromycin
40
WB: α-β-Actin
75
60
50
40
30
20
CBB
1 2 3 4 5 6 7
B
α-puromycin/α-β-Actin
(A.U.)
16
12
8
4
0
2 3 4 5 6 7 (lanes)

A

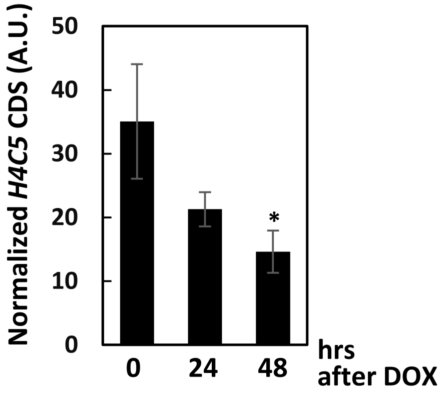
Normalized H4C5 CDS (A.U.)
50
40
30
20
10
0
*
0
24
48
hrs
after DOX

B

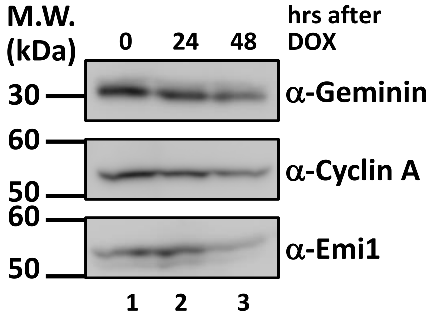
M.W.
(kDa)
0
24
48
hrs after
DOX
30
α-Geminin
60
α-Cyclin A
50
60
α-Emi1
50
1
2
3
